# Adjusting vascular permeability, leukocyte infiltration, and microglial cell activation to rescue dopaminergic neurons in rodent models of Parkinson’s disease

**DOI:** 10.1038/s41531-021-00233-3

**Published:** 2021-10-08

**Authors:** Hua-Ying Cai, Xiao-Xiao Fu, Hong Jiang, Shu Han

**Affiliations:** 1 Department of Neurology, Sir Run Run Shaw Hospital, Medical College, Zhejiang University, Hangzhou, China; 2 Institute of Anatomy and Cell Biology, Medical College, Zhejiang University, Hangzhou, China; 3 Department of Electrophysiology, Sir Run Run Shaw Hospital, Medical College, Zhejiang University, Hangzhou, China

**Keywords:** Cell biology, Cellular neuroscience

## Abstract

Animal studies have indicated that increased blood-brain barrier (BBB) permeability and inflammatory cell infiltration are involved during the progression of Parkinson’s disease (PD). This study used C16, a peptide that competitively binds to integrin α_v_β_3_ and inhibits inflammatory cell infiltration, as well as angiopoietin-1 (Ang-1), an endothelial growth factor crucial for blood vessel protection, to reduce inflammation and improve the central nervous system (CNS) microenvironment in murine models of PD. The combination of C16 and Ang-1 yielded better results compared to the individual drugs alone in terms of reducing dopaminergic neuronal apoptosis, ameliorating cognitive impairment, and electrophysiological dysfunction, attenuating inflammation in the CNS microenvironment, and improving the functional disability in PD mice or rats. These results suggest neuroprotective and anti-inflammatory properties of the C16 peptide plus Ang-1 in PD.

## Introduction

Neuroinflammation, referring to inflammation in the nervous system, is associated with many neurodegenerative diseases, such as Parkinson’s disease (PD), amyotrophic lateral sclerosis, Alzheimer’s disease, and Huntington’s disease^1^. Although inflammatory mediator-induced neurotoxicity has been recognized as a major contributor to neurodegenerative diseases, a breakdown of the blood-brain barrier (BBB) has also been implicated in PD progression^2^. BBB disruption and enhanced endothelial permeability are the hallmarks of neurovascular inflammation. Persistent inflammatory responses, involving glial cell activation and T cell infiltration, are common characteristics of PD patients and play an important role in the degeneration of dopaminergic neurons^3^.

Angiopoietin-1 (Ang-1) is an agonist ligand for the Tie-2 receptor that inhibits inflammatory cell infiltration and ameliorates inflammation-induced vessel leakage in the central nervous system (CNS)^4^. The Ang-1/Tie-2/non-receptor protein tyrosine phosphatase pathway has been shown to prevent BBB breakdown by maintaining occludin in a dephosphorylated state, providing a novel therapeutic pathway to prevent vessel leakage associated with neuro-inflammatory disorders^4^.

The transendothelial migration of leukocytes is a dynamic process, during which leukocytes initially tether to the blood vessel wall, then transiently adhere to the endothelium (the “rolling” step) before tightly attaching to the endothelial surface, and ultimately migrating through intercellular junctions to the underlying tissues^5,6^. Integrin α_ν_β_3_ is preferentially expressed in angiogenic blood vessels and allows for the interaction between leukocytes and the endothelium^5,6^. A previous study showed that the occupancy of integrin α_ν_β_3_ inhibited the binding of monocytes to intercellular adhesion molecule-1 and blocked the migration of monocytes across the endothelium^6^. Moreover, the synthetic peptide C16 (KAFDITYVRLKF), can selectively bind to integrin α_ν_β_3_ and reduce monocyte transmigration across endothelial cells^5,6^.

In this study, Ang-1, the C16 peptide, and the combination of Ang-1 and C16 were evaluated for their effects on leukocyte infiltration, vascular permeability, neuronal death, pro-inflammatory cytokine production, and functional changes in both mouse and rat models of PD^7^.

## Results

### C16 in combination with Ang-1 is superior to each drug alone

**Fig. 1:**
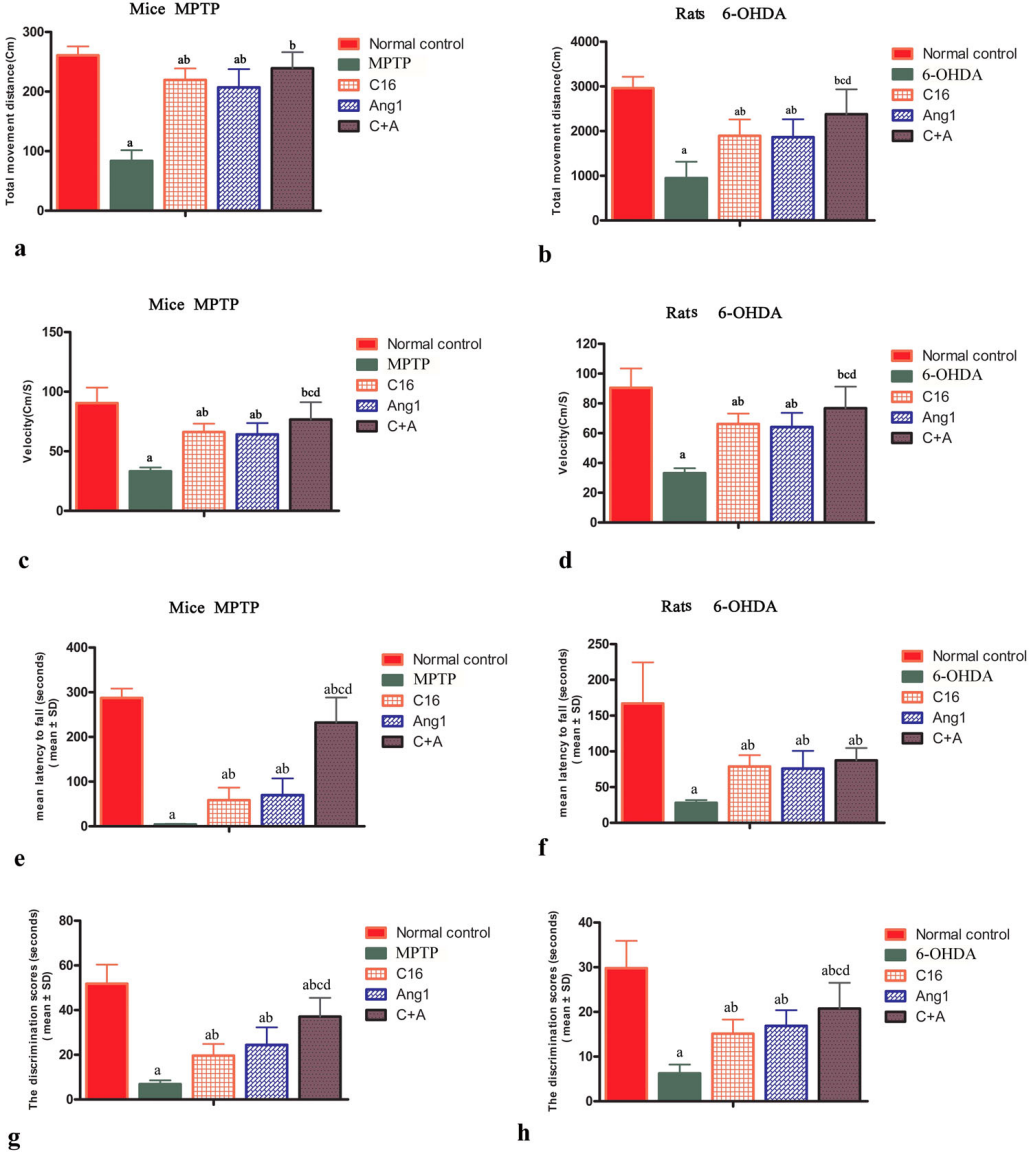
C16 in combination with Ang-1 treatment improved the functional impairment. **a**–**d** Measurements of total traveled distance (**a**: mice; **b**: rats) and mean velocity (**c**: mice; **d**: rats) of each group in the open field test. The motor function assessment revealed neurological disabilities in model animals. **a**–**f** C16 or Ang-1 alone ameliorated functional impairment in PD animals, while the combination of C16 and Ang-1 exhibited more potent therapeutic effects including the reduction in the mean velocity of both animal models in the open field test, decreased distance traveled in 6-OHDA-treated rats, and increased time on the rotarod test device in MPTP-treated mice. **g**, **h** PD animals showed an impaired ability to recognize objects, while treatment with C16 or Ang-1, and especially with C16 plus Ang-1 alleviated memory issues. **a** *p* < 0.05 versus the normal control group; **b** *p* < 0.05 versus the *p* < 0.05 versus the MPTP/6-OHDA insult group; **c** *p* < 0.05 versus the C16-treated group.; **d** *p* < 0.05 versus the Ang-1-treated group. C16, peptide (KAFDITYVRLKF) that can selectively bind integrin α_ν_β_3_; Ang-1 angiopoietin-1; PD, Parkinson’s disease.

Mice/rats exhibited functional impairment following MPTP/6-OHDA insult. In the open field test, the vehicle group showed significantly decreased traveled distance (Fig. 1a, b) and mean velocity (Fig. 1c, d) in comparison to normal controls. Also, during the rotarod test, animals in the model groups were unable to stay on the device for as long as normal controls (Fig. 1e, f). C16 or Ang-1 alone ameliorated functional impairment in PD animals, while the combination of C16 and Ang-1 exhibited more potent therapeutic effects including a reduction in the mean velocity of both animal models in the open field test, decreased distance traveled in 6-OHDA-treated rats, and increased time on the rotarod test device in MPTP-treated mice (Fig. 1a–f). In the NOR test, model animals spent similar amounts of time scrutinizing familiar and novel objects, indicating spatial cognition impairment. However, treatment with C16 or Ang-1, and especially with C16 plus Ang-1 protected animals against memory impairment (Fig. 1g, h).

### Alleviating Effects on synchronous contraction of muscles

**Fig. 2:**
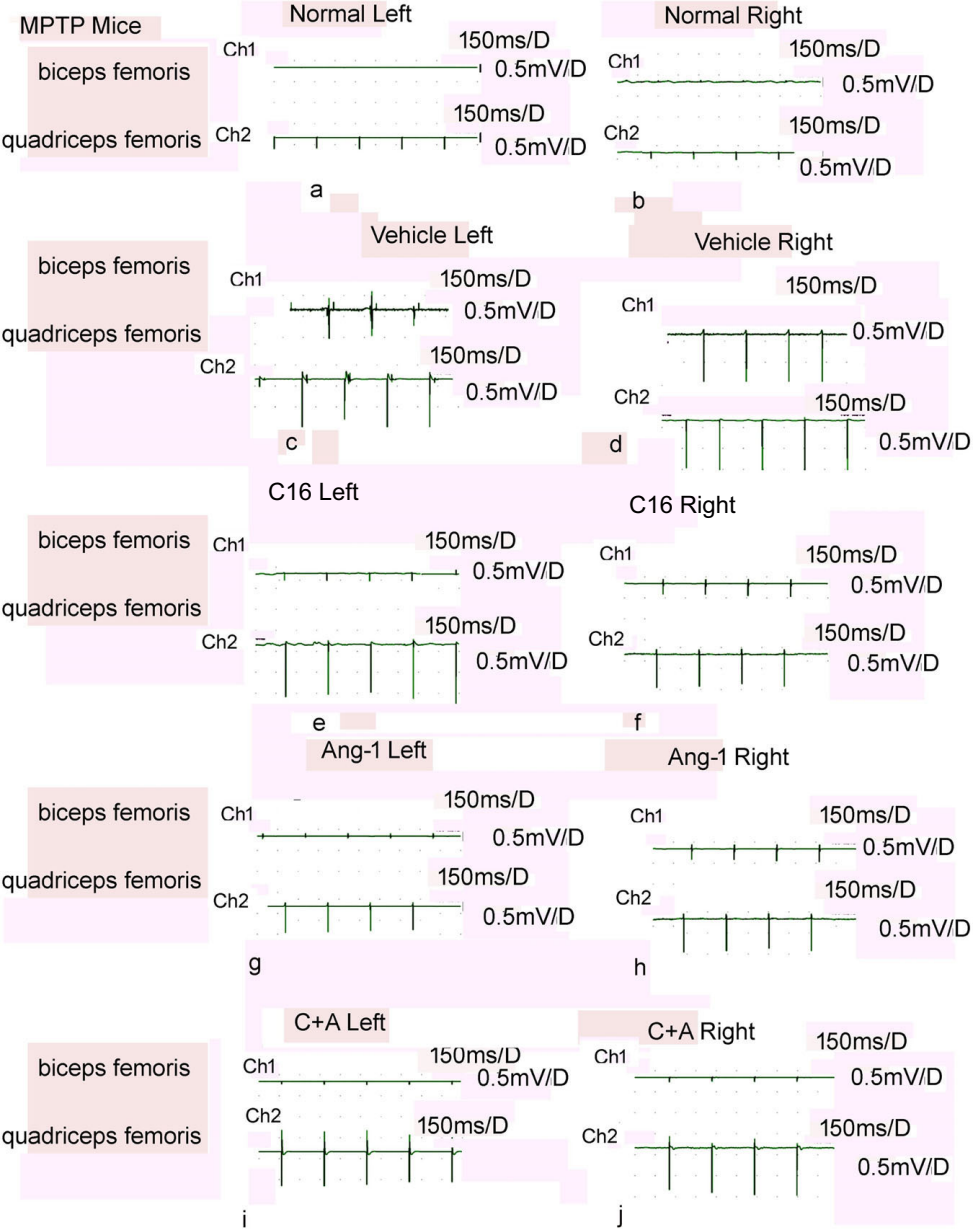
C16 in combination with Ang-1 treatment alleviated the synchronous contraction of muscles. Compared with the normal control group (**a**, **b**), MPTP-injected mice showed synchronous contraction of agonist and antagonist muscles in both lower limbs, as measured by the wave amplitude (uV) (**c**, **d**). The antagonist muscle of the model animals treated with C16 alone (**e**, **f**), Ang-1 alone (**g**, **h**), and especially the combination of C16 and Ang-1 (**i**, **j**) did not contract with the agonist muscles synchronously after stimulation. MPTP, 1-methyl-4-phenyl-1,2,3,6- tetrahydropyridine; C16, peptide (KAFDITYVRLKF) that can selectively bind integrin α_ν_β_3_; Ang-1, angiopoietin-1.

PD and dystonia are closely related disorders that share many pathophysiological overlaps. Dystonia occurs in more than 30% of PD patients^8^. It is characterized by involuntary contractions of opposing muscles, causing frequent abnormal postures or twisting movements, which could be induced in mice and rats by MPTP (Fig. 2) or 6-OHDA (Supplementary Fig. 1), respectively^9,10^. In the normal control group, the agonist muscle (quadriceps femoris) contracted, while the antagonist muscle (bicep femoris) relaxed (Fig. 2; Supplementary Fig. 1a, b). Dystonia was observed in vehicle-treated animals as evidenced by a higher uV in both muscles (mice that received systemic injections had symptoms in both hindlimbs; rats that received insult to only the right side striatum revealed symptoms in the left hindlimb) compared to the normal controls, indicating synchronous contraction of agonist and antagonist muscles in model animals (Fig. 2; Supplementary Fig. 1c, d). In animals treated with C16 (Fig. 2; Supplementary Fig. 1e, f) or Ang-1 (Fig. 2, Supplementary Fig. 1g, h), and especially in those with C16 in combination with Ang-1 (Fig. 2; Supplementary Fig. 1i, j), the antagonist muscle did not contract synchronously after stimulation.

### Suppress inflammation in the CNS

**Fig. 3:**
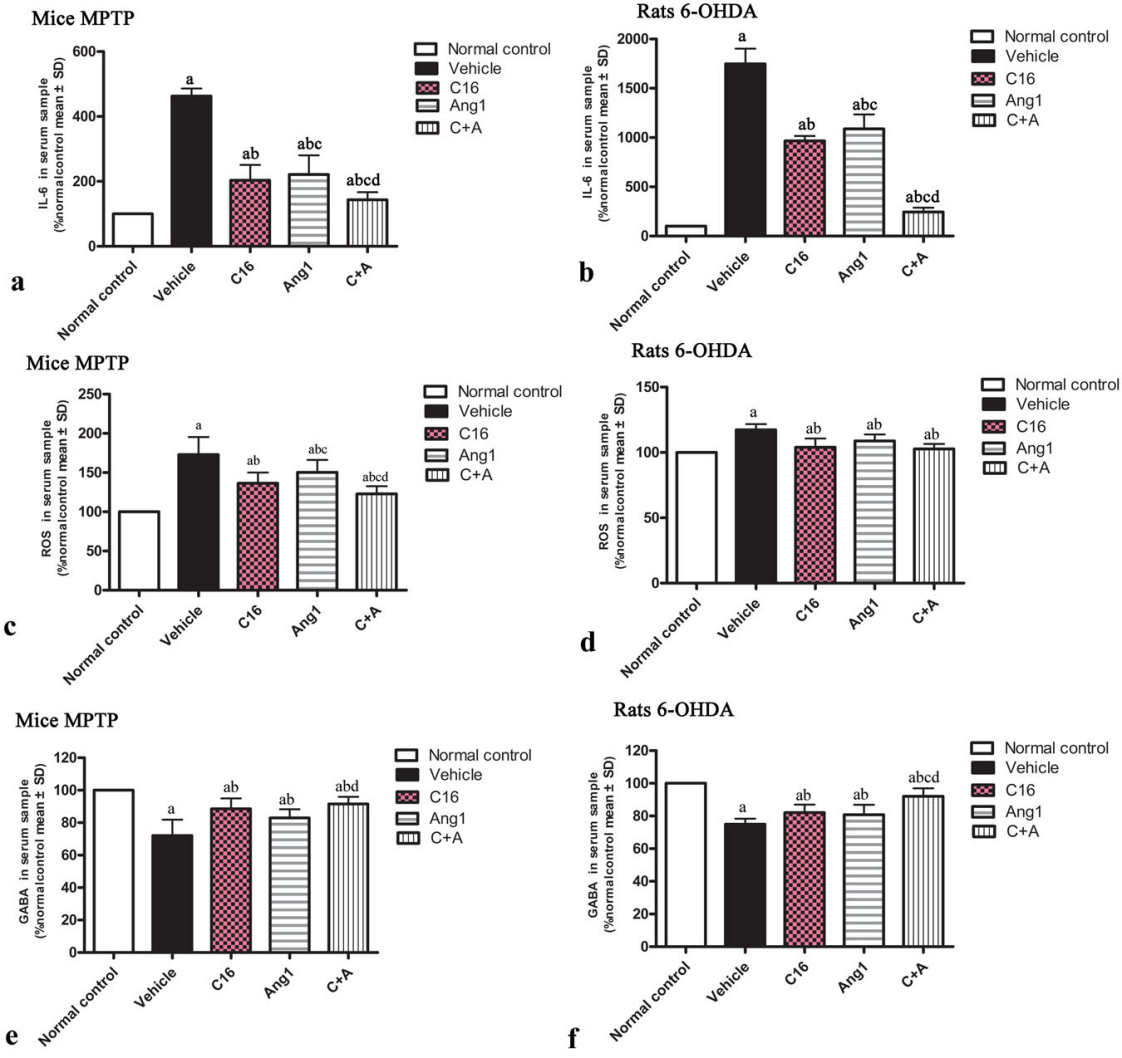
C16 in combination with Ang-1treatment reduced the expression of proinflammatory mediators. **a**–**d** Pro-inflammatory mediators, including IL-6 (**a**, **b**) and ROS (**c**, **d**) were upregulated in MPTP-injected mice (**a**, **c**) and 6-OHDA-infused rats (**b**, **d**). Treatment with C16 or Ang-1, and especially C16 plus Ang-1 significantly reduced the expression of IL-6 and ROS. **e**, **f** The expression level of GABA was decreased in MPTP-injected mice (**e**) and 6-OHDA-infused rats (**f**), but elevated following treatment with C16, Ang-1, or C16 plus Ang-1. The combined treatment showed the most potent effect. The expression of IL-6 (**a**, **b**), ROS (**c**, **d**), and GABA (**e**, **f**) in the serum samples were detected by ELISA. **a** *p* < 0.05 versus the normal control group; **b** *p* < 0.05 versus the vehicle group; **c** *p* < 0.05 versus the C16-treated group; **d** *p* < 0.05 versus the Ang-1-treated group. IL-6, interleukin-6; ROS, reactive oxygen species; MPTP, 1-methyl-4-phenyl-1,2,3,6- tetrahydropyridine; 6-OHDA, 6-hydroxydopamine; C16, peptide (KAFDITYVRLKF) that can selectively bind integrin α_ν_β_3_; Ang-1, angiopoietin-1; GABA, γ-aminobutyric acid; ELISA, enzyme-linked immunosorbent assay.

The PD model groups showed increased serum levels of IL-6 (Fig. 3a, b) and reactive oxygen species (ROS; Fig. 3c, d) compared to normal controls. The upregulation of IL-6 and ROS was significantly inhibited by treatment with C16 alone, Ang-1 alone, and C16 plus Ang-1 together (Fig. 3a, d). Furthermore, the downregulation of gamma-aminobutyric acid (GABA) following MPTP or 6-OHDA treatment in model animals was restored by C16 or Ang-1, and especially with the combination of C16 and Ang-1 (Fig. 3e, f).

**Fig. 4:**
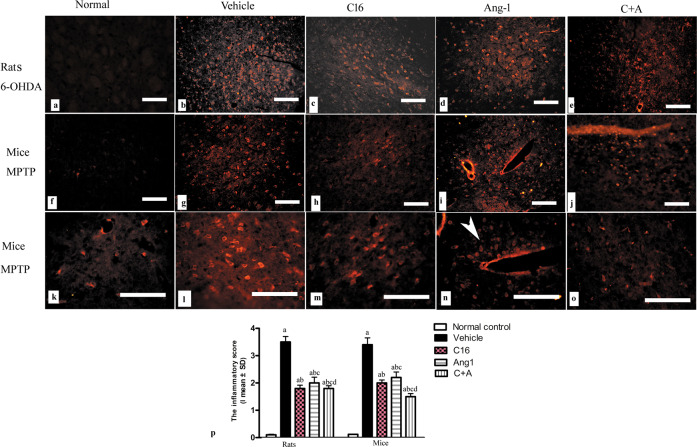
C16 in combination with Ang-1 treatment attenuated inflammatory cell infiltration. The immunostaining of CD3 revealed inflammatory cell infiltration in the vehicle-treated model group (**b**, **g**, **l**), but not in normal controls (**a**, **f**, **k**). Treatment with C16 (**c**, **h**, **m**), Ang-1 (**d**, **i**, **n**), or both (**e**, **i**, **o**) attenuated inflammation in PD animals. C16 alone or in combination with Ang-1 showed better effects than Ang-1 alone. The white arrow in N showed perivascular cuffing of CD3^+^ inflammatory cells. The inflammatory scores (**p**) were calculated. 0, no inflammation; 1, cellular infiltrates only detected around meninges and blood vessels; 2, mild infiltrates detected in parenchymal tissues (1–10 inflammatory cells per slide); 3, moderate infiltrates observed in parenchymal tissues (11–100 inflammatory cells per slide); and 4, severe infiltrates in parenchymal tissues (>100 inflammatory cells per slide). **a** *p* < 0.05 versus the normal control group; **b** *p* < 0.05 versus the vehicle group; **c** *p* < 0.05 versus the C16-treated group; **d** *p* < 0.05 versus the Ang-1-treated group. Scale bar = 100 μm. C16, peptide (KAFDITYVRLKF) that can selectively bind integrin α_ν_β_3_; Ang-1, angiopoietin-1.

Immunostaining of the pan-leukocyte marker CD-3 revealed that inflammatory cell infiltration occurred in the vehicle group (Fig. 4b, g, l), but not in normal controls (Fig. 4a, f, k). Treatment with C16 alone (Fig. 4c, h, m), Ang-1 alone (Fig. 4d, i, n), or both (Fig. 4e, i, o) attenuated inflammation in PD animals. C16 alone and in combination with Ang-1 exhibited stronger effects compared to Ang-1 alone.

**Fig. 5:**
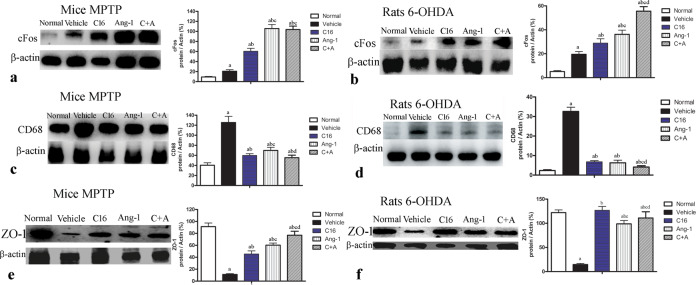
C16 in combination with Ang-1 treatment suppressed the expression of CD68 but improved the expression of cFos and ZO-1. The expression of cFos (**a**: mice; **b**: rats), CD68 (**c**: mice; **d**: rats), and ZO-1 (**e**: mice; **f**: rats) measured by western blot. Treatment with C16, Ang-1, and C16 plus Ang-1 upregulated cFos (**a**, **b**) and ZO-1, but reduced the expression of CD68 (**c**, **d**) in both mouse and rat PD models. **a** *p* < 0.05 versus the normal control group; **b** *p* < 0.05 versus the vehicle group; **c** *p* < 0.05 versus the C16-treated group; **d** *p* < 0.05 versus the Ang-1-treated group. cFos protein: 41 kDa; CD68 protein: 37 kDa; ZO-1 protein: 220 kDa; β-actin protein: 42 kDa; ZO-1, zonula occludens-1; C16, peptide (KAFDITYVRLKF) that can selectively bind integrin α_ν_β_3_; Ang-1, angiopoietin-1; PD, Parkinson’s disease.

Furthermore, the expression of the macrophage-specific marker CD68 was increased in both mouse and rat PD models, while treatment with Ang-1 alone, C16 alone, and C16 plus Ang-1 effectively repressed the upregulation of CD68, with the most robust effects observed in the C16 plus Ang-1 group (Fig. 5c, d).

The expression of the leucine-rich-repeat kinase 2 *(LRRK2)* gene is biochemically associated with pathways that regulate inflammation, autophagy, and phagocytosis^11^. The immunostaining of LRRK2^+^ cells and western blot analysis in striatum showed that LRRK2 was upregulated in vehicle-treated PD models compared to normal controls, but was inhibited by treatment with C16, Ang-1, and the combination of C16 and Ang-1 (Supplementary Fig. 2).

Moreover, western blot analysis revealed that the upregulation of pro-inflammatory factors nuclear-factor kappa B (NF-κB; Supplementary Fig. 3a, mice; b, rats) and cyclooxygenase-2 (COX-2; Supplementary Fig. 3c mice; d, rats) in vehicle-treated PD models was inhibited by Ang-1 alone, C16 alone, as well as C16 plus Ang-1.

### Reduce blood vessel leakage and BBB permeability

Severe vasculature leakage was noted in vehicle-treated mice and rats (Supplementary Fig. 4b, l). The expression of ZO-1, a tight junction protein between endothelial cells lining blood vessels, was downregulated (Fig. 5e, f; Supplementary Fig. 4g, q) compared to the normal controls (Fig. 5e, f; Supplementary Fig. 4a, f, k, p). However, PD animals treated with C16 (Supplementary Fig. 4c, h, m, r), Ang-1 (Supplementary Fig. 4d, i, n, s), and especially the combination of C16 and Ang-1 (Supplementary Fig. 4e, j, o, t) showed greatly reduced leakage from blood vessels (Supplementary Fig. 4u). The expression of ZO-1 was also increased in PD mice/rats treated with C16 or Ang-1, and especially with the combination of C16 with Ang-1 (Fig. 5e, f; Supplementary Fig. 4v).

**Fig. 6:**
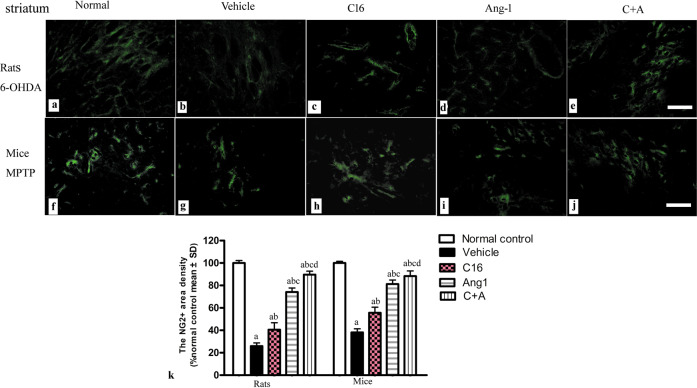
C16 in combination with Ang-1 treatment restored the expression of NG2. **a**–**j** The immunostaining of NG2^+^ pericytes (green) in the striatum showed that the expression of NG2 was decreased in vehicle-treated PD models (**b**, **g**), but restored by C16 (**c**, **h**), Ang-1 (**d**, **i**), and especially the C + A treatment (**e**, **j**). Scale bar = 100 µm. **k** Quantification of the immunostaining of NG2^+^ pericytes. **a** *p* < 0.05 versus the normal control group; **b** *p* < 0.05 versus the vehicle group; **c** *p* < 0.05 versus the C16-treated group; **d** *p* < 0.05 versus the Ang-1-treated group. NG2, neural/glial antigen 2 proteoglycan; C16, peptide (KAFDITYVRLKF) that can selectively bind integrin α_ν_β_3_; Ang-1 angiopoietin-1; PD, Parkinson’s disease.

The BBB constitutes a neurovascular unit formed by microvascular endothelial cells, pericytes, and astrocytes. The proteoglycan neural/glial antigen 2 (NG2) plays a key role in proliferation, migration, and differentiation of pericytes and oligodendroglial precursor cells in the brain^12^. The immunostaining of NG2^+^ pericytes in the striatum revealed that the downregulation of NG2 in PD models (Fig. 6b, g) was restored by treatment with C16 (Fig. 6c, h), Ang-1 (Fig. 6d, i), and especially the combination of C16 and Ang-1 (Fig. 6e, j).

**Fig. 7:**
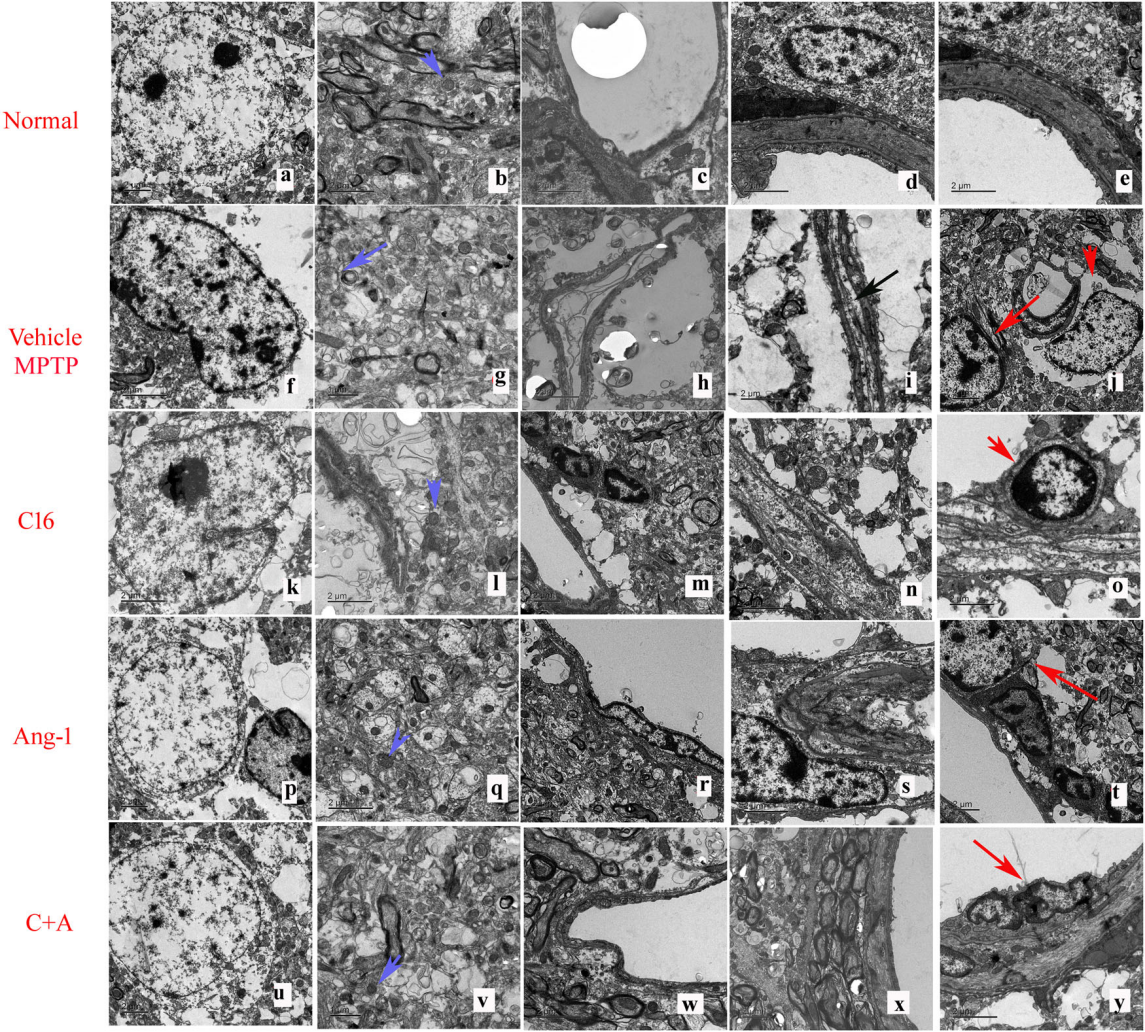
Electron microscopy revealed the ultrastructural morphology of the striatum in each group. Scale bar = 2 μm. normal neuronal nuclei with uncondensed chromatin (**a**). Normal myelinated axons with dark, ring-shaped myelin sheaths surrounding axons, and typically shaped mitochondria with clear cristae (blue arrow in **b**). The normal control group showed no tissue edema or blood vessel leakage (**c**) but had intact tight junctions (**d**). No inflammatory cell infiltrate was found in normal tissues (**e**). The neurons in the vehicle-treated PD model group had shrunken nuclei with condensed, fragmented, and marginated nuclear chromatin (**f**). mitochondria vacuolization with swollen cristae (blue arrow in **g**). Severe vascular vessel leakage and tissue edema were detected in the extracellular space surrounding blood vessels (**h**). The tight junctions between the endothelial cells were lost (black arrow in **i**). Inflammatory cell infiltration was observed surrounding blood vessels (red arrow in **j**). However, in C16 (**k**–**o**), Ang-1 (**p**–**t**), and C16 plus Ang-1 treated groups (**u**–**y**), the morphology of the nuclei (**k**, **p**, **u**), myelin, and axons (**l**, **q**, **v**) were relatively normal. Perivascular edema (**m**, **r**, **w**) was alleviated. The morphology of the tight junctions (**n**, **s**, **x**) was also relatively normal, especially in the groups treated with Ang-1 alone or in combination with C16 (**s**, **x**). The morphology of the mitochondria was relatively normal (blue arrows in **l**, **q**, and **v**). Inflammatory cell infiltration (red arrows in **o**, **t**, **y**) was mainly detected within blood vessels in the C16 alone and C16 plus Ang-1 groups (**o**, **y**), and was reduced in Ang-1-treated group (**t**) when compared with the model animals (**j**). PD, Parkinson’s disease; C16, peptide (KAFDITYVRLKF) that can selectively bind integrin α_ν_β_3_; Ang-1, angiopoietin-1.

Electron microscopy showed the ultrastructural morphology of the striatum in each group. In the normal group, electron microscopy revealed normal neuronal nuclei with uncondensed chromatin (Fig. 7a), normal myelinated axons with dark, ring-shaped myelin sheaths surrounding axons, and typically shaped mitochondria with clear cristae (blue arrow in Fig. 7b). The normal group also showed intact tight junctions and had no tissue edema, blood vessel leakage, or inflammatory cell infiltration (Fig. 7c–e). In vehicle-treated PD model groups, apoptotic neurons with shrunken nuclei and fragmented, condensed, and marginated nuclear chromatin were detected (Fig. 7f). The myelin sheaths identified in model animals were fused, splitting, and loose with vacuoles, and the mitochondria showed vacuolization with swollen cristae (blue arrow in Fig. 7g). Tissue edema and severe blood vessel leakage were also detected in the extracellular space surrounding blood vessels (Fig. 7h). The tight junctions between the endothelial cells were lost (black arrow in Fig. 7i) and inflammatory cell infiltration was found surrounding blood vessels (red arrow in Fig. 7j). However, in the groups treated with C16 (Fig. 7k–o) or Ang-1 (Fig. 7p–t) alone, as well as C16 plus Ang-1 (Fig. 7u–y), the morphology of the nuclei (Fig. 7k, p, u), myelin, and axons (Fig. 7l, q, v) were relatively normal. In addition, perivascular edema (Fig. 7m, r, w) was alleviated and the morphology of tight junctions (Fig. 7n, s, x) was relatively normal, especially in the groups treated with Ang-1 alone or in combination with C16 (Fig. 7s, x). The morphology of the mitochondria was also relatively normal (blue arrows in Fig. 7l, q and w). In model animals treated with C16 and C16 plus Ang-1, inflammatory cells were mainly detected within blood vessels (red arrows in Fig. 7o, y). Inflammatory cell infiltration was also reduced in the Ang-1-treated group (Fig. 7t) in comparison to vehicle-treated model animals (arrows in Fig. 7j).

### Rescue neurons from death and suppress activated astrocytes

**Fig. 8:**
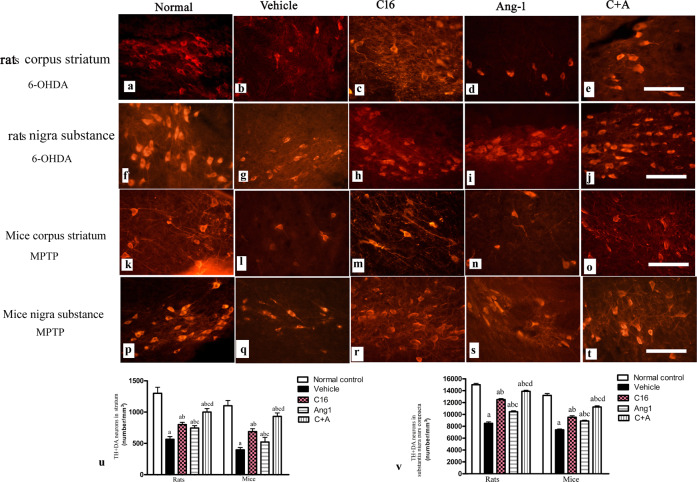
C16 in combination with Ang-1 treatment rescued the TH neurons. **a**–**t** The DA neurons were stained using anti-TH (red) in both the nigra substance and striatum of PD rats (induced by 6-OHDA) and PD mice (induced by MPTP). Treatment with C16 (**c**, **h**, **m**, **r**), Ang-1 (**d**, **i**, **n**, **s**), and especially the combination of C16 and Ang-1 (**e**, **j**, **o**, **t**) reduced DA neuron loss in PD models (**b**, **g**, **l**, **q**). Panel **a**, **f**, **k**, **p** show DA neurons in normal controls. Scale bar = 100 µm. **u**, **v** Stereological analysis of DA neurons in the corpus striatum (**u**) and SN (**v**). **a** *p* < 0.05 versus the normal control group; **b** *p* < 0.05 versus the vehicle group; **c** *p* < 0.05 versus the C16-treated group; **d** *p* < 0.05 versus the Ang-1-treated group. DA, dopamine; TH, tyrosine hydroxylase; GABA, gamma-aminobutyric acid; MPTP, 1-methyl-4-phenyl-1,2,3,6-tetrahydropyridine; 6-OHDA, 6-hydroxydopamine; C16, peptide (KAFDITYVRLKF) that can selectively bind integrin α_ν_β_3_; Ang-1 angiopoietin-1; PD, Parkinson’s disease.

**Fig. 9:**
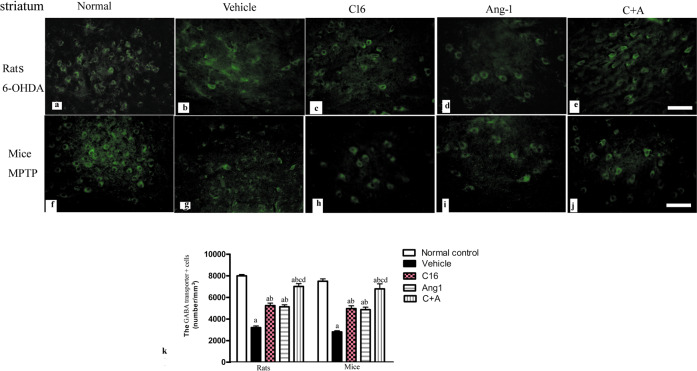
C16 in combination with Ang-1 treatment rescued the GABA neurons. **a**–**j** The immunostaining of GABA transporter-positive cells (green) in the striatum showed that activated GABA was downregulated in vehicle-treated PD models compared to the normal control group (**b**, **g**). Treatment with C16 (**c**, **h**), Ang-1 (**d**, **i**), and the C16 plus Ang-1 treatment (**e**, **j**) restored GABA expression in PD animals. Scale bar = 100 µm. **k** Quantification of cells stained with anti-GABA transporter. **a** *p* < 0.05 versus the normal control group; **b** *p* < 0.05 versus the vehicle group; **c** *p* < 0.05 versus the C16-treated group; **d** *p* < 0.05 versus the Ang-1-treated group. GABA, gamma-aminobutyric acid; C16, peptide (KAFDITYVRLKF) that can selectively bind integrin α_ν_β_3_; Ang-1 angiopoietin-1.

**Fig. 10:**
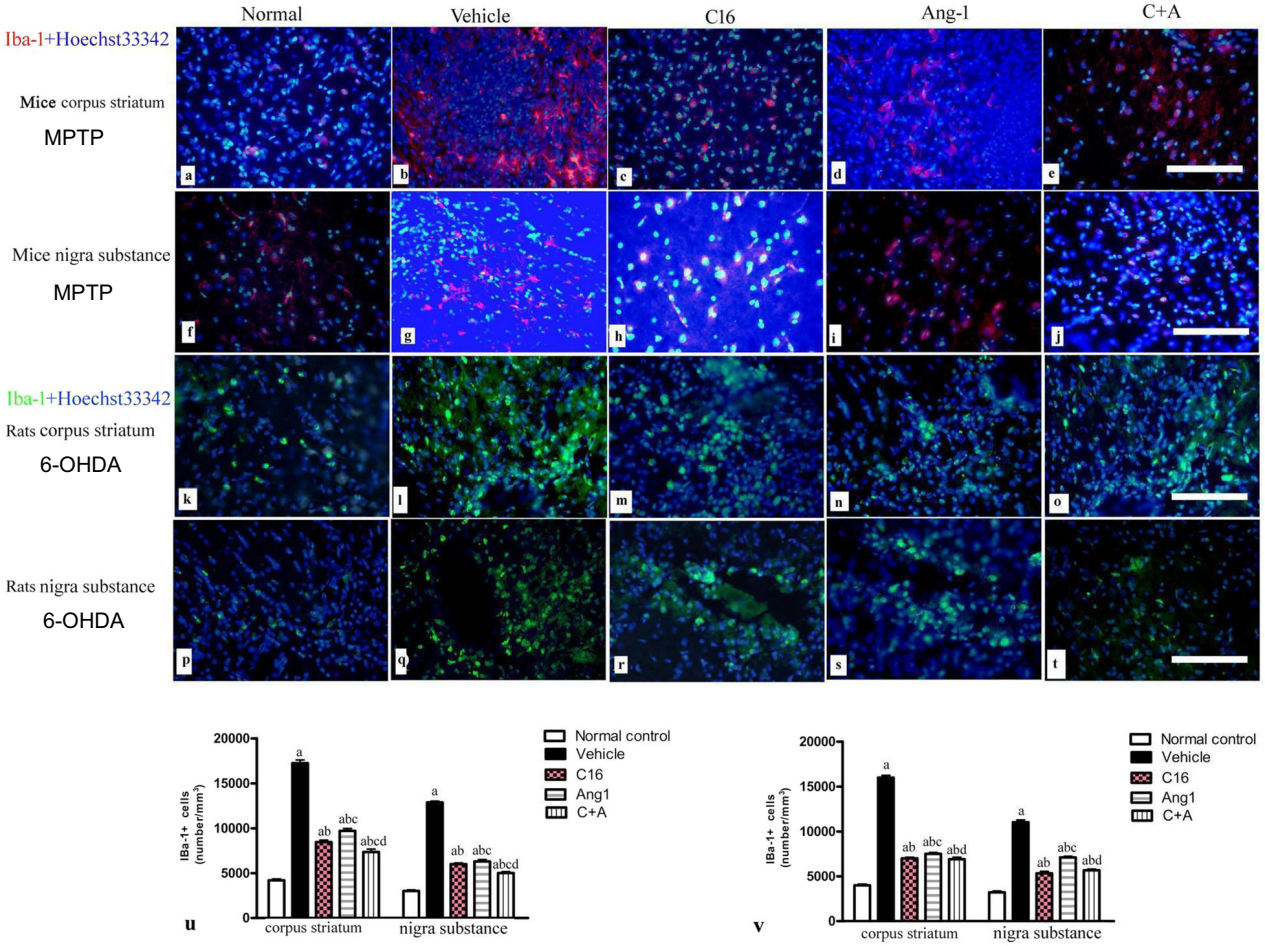
C16 in combination with Ang-1 treatment supressed microglia activation. **a**–**l** The immunostaining of Iba-1^+^ cells (red in mice and green in rats) in the corpus striatum and nigra substance showed that C16 (**c**, **h**, **m**, **r**), Ang-1 (**d**, **i**, **n**, **s**), and especially the C16 plus Ang-1 treatment (**e**, **j**, **o**, **t**) suppressed the increase of microglia in PD models (**b**, **g**, **l**, **q**). Panel **a**, **f**, **k**, **p** show the normal control group. The nuclei of cells were labeled with Hoechst33342 (blue). Scale bar = 100 µm. (**u**, **v**): Stereological analysis of Iba-1^+^ cells in mice (**u**) and rats (**v**). (**a**), *p* < 0.05 versus the normal control group; (**b**), *p* < 0.05 versus the vehicle group (**c**), *p* < 0.05 versus the C16-treated group (**d**), *p* < 0.05 versus the Ang-1-treated group. Abbreviations: Iba-1, ionized calcium binding adapter molecule 1; C16, peptide (KAFDITYVRLKF) that can selectively bind integrin α_ν_β_3_; Ang-1 angiopoietin-1.

**Fig. 11:**
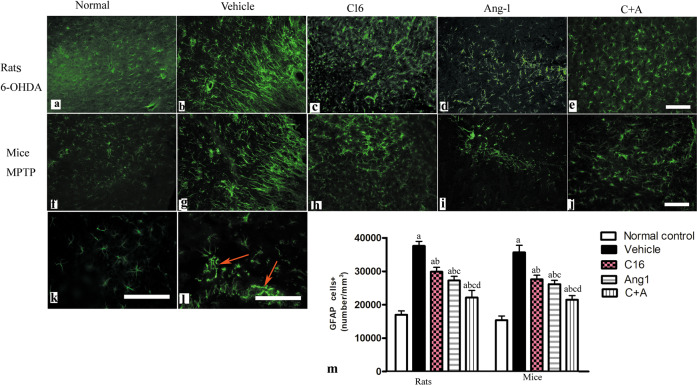
C16 in combination with Ang-1 treatment supressed the activated astrocytes. **a**–**l** The activated astrocytes in the SN were stained with GFAP (green). C16 (**c**, **h**), Ang-1 (**d**, **i**), and especially the C + A treatment (**e**, **j**) suppressed the proliferation of astrocytes in vehicle-treated PD models (**b**, **g**). Panel **k** shows astrocytes in the normal control group. Panel **l** shows the proliferated glial cells and glial scar (red arrow in **l**) in the vehicle-treated group in high magnification. Scale bar = 100 µm. **m** Stereological analysis of GFAP + cells. **a** *p* < 0.05 versus the normal control group; **b** *p* < 0.05 versus the vehicle group; **c** *p* < 0.05 versus the C16-treated group; **d** *p* < 0.05 versus the Ang-1-treated group. GFAP, glial fibrillary acidic protein; C16, peptide (KAFDITYVRLKF) that can selectively bind integrin α_ν_β_3_; Ang-1 angiopoietin-1.

Next, a stereological analysis of neurons was performed. The immunostaining of dopamine (DA) neurons (by tyrosine hydroxylase [TH]), astrocytes (by glial fibrillary acidic protein [GFAP]), and microglia (by ionized calcium binding adapter molecule 1 [Iba-1]) in the substantia nigra, and DA neurons, medium spiny neurons (by GABA transporter), and microglia in the striatum showed that C16, Ang-1, and especially the combination of C16 and Ang-1 protected DA neurons in the nigra substance (Fig. 8) and GABA transporter-positive neurons in the corpus striatum (Fig. 9). These treatments also reduced the loss of CHAT^+^ neurons in both the nigra substance and striatum of vehicle-treated PD models (Supplementary Fig. 5). Furthermore, the counting of microglia/macrophages (identified by Iba-1, Fig. 10) and activated astrocytes (identified by GFAP) suggested that C16, Ang-1, and the combination of C16 and Ang-1 reduced the number of microglia, suppressed activated astrocytes, and inhibited the formation of glial scars (arrow in Fig. 11l) in PD models.

Additionally, the expression of both DA (Supplementary Fig. 4g, mice; h, rats) and GABA (Supplementary Fig. 4i, mice; j, rats) was decreased in vehicle-treated PD models. However, Ang-1, C16, and C16 plus Ang-1 restored the expressions of these proteins.

**Fig. 12:**
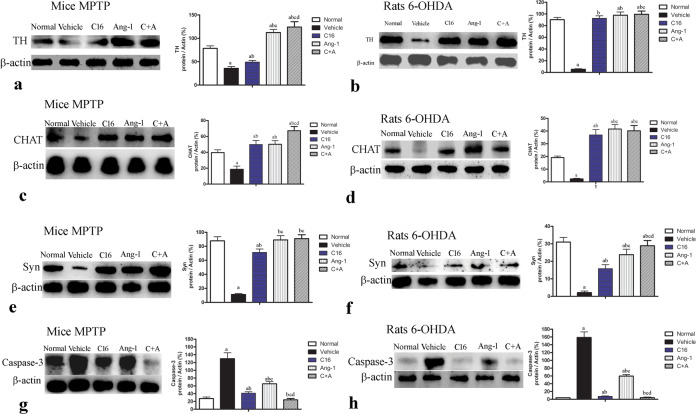
C16 in combination with Ang-1 treatment improved the expression of TH, CHAT, Syn but inhibited the expression of active caspase-3. The expression of TH (**a**: mice; **b**: rats), CHAT (**c**: mice; **d**: rats), Syn (**e**: mice; **f**: rats), and active caspase-3 (**g**: mice; **h**: rats) were measured by western blot. Treatment with C16, Ang-1, and C16 plus Ang-1 upregulated TH, CHAT and Syn, but reduced the expression of active caspase-3 in both mouse and rat PD models. **a** *p* < 0.05 versus the normal control group; **b** *p* < 0.05 versus the vehicle group; **c** *p* < 0.05 versus the C16-treated group; **d** *p* < 0.05 versus the Ang-1-treated group. TH protein: 58 kDa; CHAT protein: 82 kDa; Syn protein: 34 kDa; Caspase-3 protein: 32 kDa; β-actin protein: 42 kDa; TH, tyrosine hydroxylase; CHAT, choline acetyltransferase; Syn, synaptophysin; C16, peptide (KAFDITYVRLKF) that can selectively bind integrin ανβ3; Ang-1, angiopoietin-1.

**Fig. 13:**
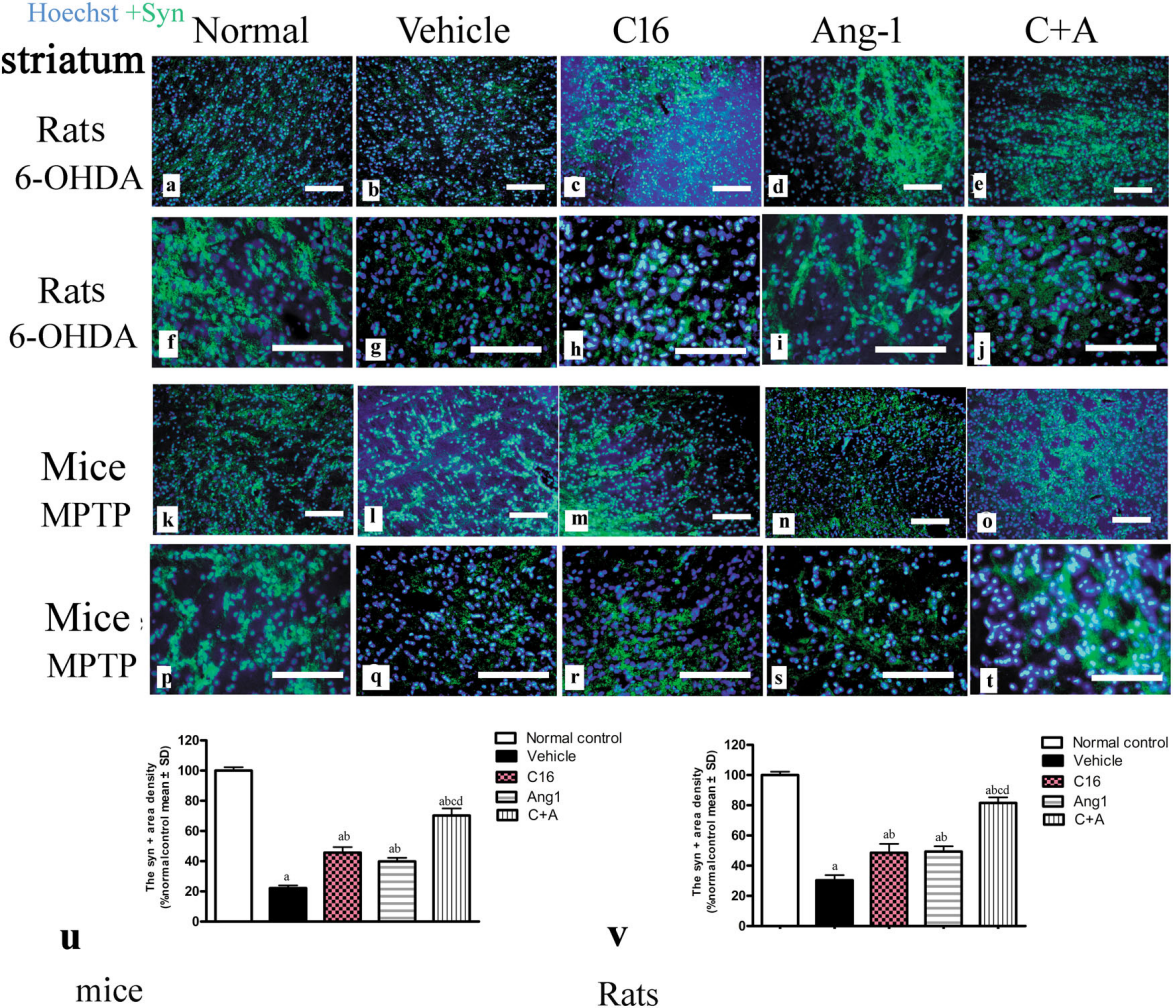
Syn-positive neurons were detected by immunofluorescence staining. The model animals showed a notably decreased number of Syn-positive cells in the corpus striatum and nigra substance (**a**–**j** rats; **k**–**t** mice), while treatment with C16, Ang-1, and especially C16 plus Ang-1 remarkably reversed this phenomenon in both models (**u**, **v**). The nuclei of cells were stained using Hoechst 33342 (blue). **a** *p* < 0.05 versus the normal control group; **b** *p* < 0.05 versus the vehicle group; **c** *p* < 0.05 versus the C16-treated group; **d** *p* < 0.05 versus the Ang-1-treated group. Scale bar = 100 μm. Syn, synaptophysin; C16, peptide (KAFDITYVRLKF) that can selectively bind integrin α_ν_β_3_; Ang-1, angiopoietin-1.

In both models of PD, western blot analysis of the corpus striatum and nigra substance revealed that treatment with C16, Ang-1, and C16 plus Ang-1 restored the expressions of TH (Fig. 12a, b), CHAT (Fig. 12c, d), and Syn (Fig. 12e, f), and inhibited the expression of active caspase-3 (Fig. 12g, h), an enzyme involved in apoptotic cell death. Immunofluorescent staining of TH (Fig. 8), GABA transporter (Fig. 9) CHAT (Supplementary Fig. 5), and Syn, a synapse-associated protein that promotes synaptic plasticity (Fig. 13), also confirmed these changes. The drug-treated groups also showed less staining for active caspase-3 (Supplementary Fig. 6) and LC3 (Supplementary Fig. 7), a marker of autophagy, in the corpus striatum and nigra substance compared to model animals. The largest difference was observed in the C16 plus Ang-1 group.

**Fig. 14:**
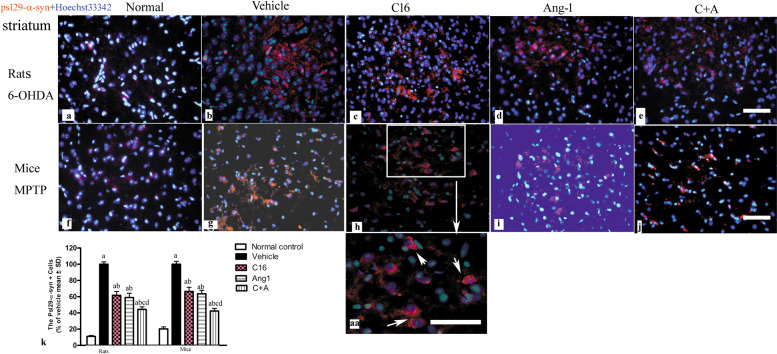
The pS129-a-synpositive cells were detected by immunofluorescence staining. **a**–**j** The immunostaining of pS129-α-syn^+^ cells (red) in the striatum showed that the expression of pS129-α-syn^+^ was increased in vehicle-treated PD models (**b**, **g**) compared to normal controls (**a**, **f**). Treatment with C16 (**c**, **h**), Ang-1 (**d**, **i**), and especially C16 plus Ang-1 (**e**, **j**) inhibited the upregulation of pS129-α-syn^+^ in PD animals. Image “aa” shows the enlarged image within the square frame of (**h**). The white arrow indicates pS129-α-syn^+^ staining. The nuclei of cells were labeled with Hoechst33342 (blue). Scale bar = 100 µm. **k** Quantification of pS129-α-syn^+^ cells. **a** *p* < 0.05 versus the normal control group; **b** *p* < 0.05 versus the vehicle group; **c** *p* < 0.05 versus the C16-treated group; **d** *p* < 0.05 versus the Ang-1-treated group. pS129-α-synuclein, plasma Ser129-phosphorylated α-synuclein; C16, peptide (KAFDITYVRLKF) that can selectively bind integrin ανβ3; Ang-1, angiopoietin-1.

pS129-α-syn is a surrogate marker of PD progression^13^. The vehicle-treated PD models (Fig. 14b, g) showed an increased number of pS129-α-syn^+^ cells compared to normal controls (Fig. 14a, f). However, treatment with C16 (Fig. 14c, h), Ang-1 (Fig. 14d, i), and especially the combination of C16 plus Ang-1 (Fig. 14e, j) inhibited the expression of pS129-α-syn in PD animals. Western blot analysis also confirmed these changes (Supplementary Fig. 4e, mice; f, rats).

### Regulate expression of factors involved in several pathways

Following systemic injection of MPTP or regional infusion of 6-OHDA into the right corpus striatum, mice/rats showed increased expression of the immediate early gene cFos compared to normal controls (Fig. 5a, b; Supplementary Fig. 8), while treatment with C16, Ang-1, and C16 plus Ang-1 further induced the expression of cFos (Fig. 5a, b; Supplementary Fig. 8).

**Fig. 15:**
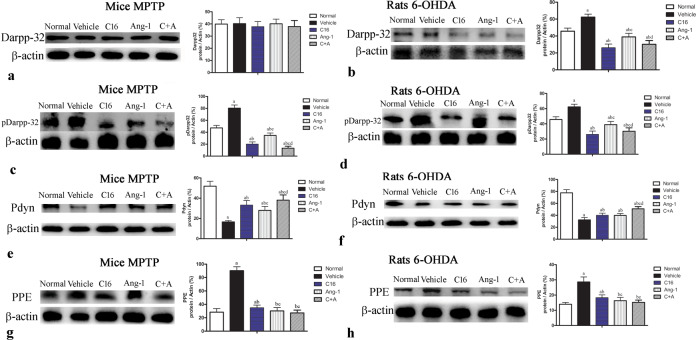
C16 in combination with Ang-1 treatment decreased the expression of phospho-DARPP-32, and increased the expression of pdyn and PPE. Western blot analysis showed no significant difference in the expression of DARPP-32 among the different groups (**a**, **b**). The expression of phospho-DARPP-32 in the vehicle-treated group was higher than that in normal controls (**c**, **d**). The combination of C16 and Ang-1 inhibited the upregulation of phospho-DARPP-32 in model animals. The downregulation of prodynorphin (pdyn, **e**, **f**) and the upregulation of preproenkephalin (PPE, **g**, **h**) in the model groups were remarkably reversed by treatment with C16, Ang-1, and C16 plus Ang-1. **a** *p* < 0.05 versus the normal control group; **b** *p* < 0.05 versus the vehicle group (**c**), *p* < 0.05 versus the C16-treated group (**d**), *p* < 0.05 versus the Ang-1-treated group. Darpp-32 protein: 34 kDa; pDarpp-32 protein: 34 kDa; Pdyn protein: 28 kDa; PPE protein: 30 kDa; β-actin protein: 42 kDa; Abbreviations: DARPP, dopamine- and cAMP-regulated neuronal phosphoprotein; pdyn, prodynorphin; PPE, preproenkephalin; C16, peptide (KAFDITYVRLKF) that can selectively bind integrin α_ν_β_3_; Ang-1, angiopoietin-1.

There was no significant difference in DARPP-32 expression level between the groups (Fig. 15a, b). However, the model groups showed a significantly higher level of phospho-DARPP-32 compared to normal controls, while C16 alone, Ang-1 alone, and especially C16 plus Ang-1 remarkably suppressed the expression phospho-DARPP-32 in PD model animals (Fig. 15c, d, Supplementary Fig. 9).

The expressions of preproenkephalin and prodynorphin, two indicators of imbalance between the striatopallidal and striatonigral pathways, were also examined. In the corpus striatum and nigra substance of the vehicle-treated model animals, the basal expression of prodynorphin was reduced (Fig. 15e, f, Supplementary Fig. 10), while that of preproenkephalin was tending toward an increase in comparison to normal controls (Fig. 15g, h; Supplementary Fig. 11). However, treatment with C16 alone, Ang-1 alone, and the combination of C16 plus Ang-1 effectively reversed these alterations in expression (Fig. 15e–h; Supplementary Figs. 10, 11).

## Discussion

Oxidative stress-induced mitochondrial dysfunction and damage play a fundamental role in PD. Oxidative stress leads to excessive production of ROS, which chemically interact with biological molecules, resulting in functional changes in cells and ultimately cell death. Importantly, neuroinflammation and mitochondrial dysfunction are common characteristics of PD^14^.

Models using toxins represent the classic experimental PD models and aim to reproduce pathological and behavioral changes observed in PD patients via local or systemic administration of animals with neurotoxins that selectively induce nigrostriatal neuron degeneration^15^. A typical systemic PD model is established by the injection of MPTP, an agent with selective toxicity for dopaminergic neurons. After crossing the BBB, MPTP is transformed into its active metabolite by monoamine oxidase B and then carried by the dopamine transporter (DAT) into the dopaminergic neurons of the substantia nigra pars compacta (SNpc), where it blocks the activity of mitochondrial complex I^15^. The MPTP-induced inflammatory response, neuronal apoptosis, and oxidative stress in the brain is essential for the occurrence of the chronic inflammatory reaction in mice^16^. As rats are highly resistant to MPTP toxicity, mice are commonly used to establish the MPTP model of PD^15^.

The rat model of PD is established by local (i.e., intracerebral) injection of 6-OHDA, a hydroxylated analog of dopamine with high affinity for the DAT and has been shown to transport toxins into dopaminergic neurons^14,15^. The injection of 6-OHDA into the striatum causes prompt damage of striatal terminals and significant anterograde degeneration of the nigrostriatal pathway, followed by the progressive loss of SNpc neurons. The pro-oxidant property of 6-OHDA is responsible for the damage in neurons. Once inside the neurons, 6-OHDA undergoes auto-oxidation in the cytosol, which leads to the formation of hydrogen peroxide. Meanwhile, 6-OHDA also accumulates in the mitochondria, where it inhibits the activity of mitochondrial complex I^14,15^.

The degeneration of dopaminergic neurons located in the pars compacta of the substantia nigra that project to the striatum (the nigrostriatal pathway) is a typical neuropathological feature of PD. The cardinal motor signs of PD are tremors, rigidity, slow movement (bradykinesia), poor balance, and difficulty walking (Parkinsonian gait). The non-motor symptoms of PD include autonomic, psychiatric, cognitive impairments^16^. GABA interneurons, the main inhibitory neurons in the CNS, play a critical role in a multitude of physiological processes, including modulation of cortical and hippocampal neural circuitry and activity^17^. In this study, the behavior tests detected the impairment of both motor function and cognitive ability, which were in accordance with the loss of dopaminergic and GABA neurons in the substantia nigra and striatum.

The degeneration of dopaminergic and GABA neurons in PD is related to oxidative stress and inflammation^17^. The abnormal upregulation of the inflammatory cytokine IL-6 in the serum and high expression of NF-κB and COX-2 in the nigra and striatum of vehicle-treated PD animals supported these previous findings. NF-κB is a transcription factor regulated by oxidative stress. It plays a key role in the activation of inflammatory responses. COX-2 is also involved in the production of pro-inflammatory prostaglandins. The upregulation of these pro-inflammatory factors in the vehicle-treated groups implied that inflammatory responses were triggered by oxidative stress^18^. Agents with anti-inflammatory properties have been shown to reduce the risk or slow the progression of PD^19^. Leukocyte infiltration into the brain is a key contributor to local inflammation and dopaminergic neuron death after MPTP exposure^20^. Also, reduced inflammation may lead to better outcomes for late-stage neurodegeneration^21^. Recent evidence suggests that inflammation plays a conclusive role in the neurodegenerative process of PD^22^. In healthy brains, microglia display a resting phenotype and act as scavengers to remove waste products and debris from the parenchyma. However, the accumulation of toxins, tissue damage, infection, or other trigger molecules can activate microglial cells, which then become the major source of ROS during neuro-inflammatory reactions^23^. Dopamine auto-oxidation-related neurodegeneration is the main mechanism underlying PD. Activated microglia (as shown by increased Iba-1 expression in the vehicle-treated group) exacerbate inflammatory responses by upregulating the production of chemokines that recruit immune cells to the CNS and pro-inflammatory cytokines that cause neurotoxicity^24^. Resulting from the release of chemo-attractants, the self-perpetuating cycle of microglial activation further promotes activation of the microglia and neuroinflammation^22^.

A previous study showed that intravenous administration of Ang-1 and C16 induced secondary tissue damage through the regulation of vascular events, such as leukocyte extravasation (as demonstrated by the staining of the pan-leukocyte marker CD-3) and endothelial cell death^6^.

Ang-1 acts through Tie-2, which is solely expressed in endothelial cells, to rescue blood vessels. It also reduces vascular permeability under pathological conditions by preserving the integrity of endothelial tight junctions. Moreover, Ang-1 reduced inflammation in vivo by inhibiting microglia/macrophage activation and infiltration, as confirmed by the remarkably decreased number of Iba-1-labeled cells^6^.

The C16 peptide promotes endothelial cell survival by activating α_5_β_1_ and α_v_β_3_ integrins. It also reduces leukocyte transmigration across the endothelium by binding and blocking the α_v_β_3_ integrin^23^. The inhibition of inflammatory cell activation and infiltration also reduces the release of chemo-attractants in the microenvironment of the CNS. Although the function of C16 partially overlaps with that of Ang-1, the combination of C16 and Ang-1 has been confirmed to have a synergistic effect in suppressing inflammation in the CNS, as reported in our previous studies^24–28^.

The expression of LRRK2 in microglia is upregulated upon activation^29,30^. Moreover, LRRK2 is associated with pathways that regulate inflammation, autophagy, and phagocytosis. Increasing evidence suggests that inflammation in the CNS plays an important role in PD pathophysiology^11^. LRRK2 inhibition has been shown to prevent the loss of dopaminergic neurons and motor deficits induced by lipopolysaccharide^31^. Furthermore, LRRK2 deletion or pharmacological inhibition decreased the production of pro-inflammatory mediators (i.e., IL-1β and COX-2) and subsequently reduced inflammatory responses^32^. In this study, vehicle-treated PD models, in which microglia were challenged, showed upregulation of LRRK2 expression, but this phenomenon was inhibited by C16, Ang-1, and especially the combination therapy, which may be due to the decreased number of inflammatory cells caused by these treatments.

The synapse-associated proteins, especially presynaptic Syn, can promote synaptic plasticity. Syn deficiency is correlated with cognitive decline in aging-related neurodegenerative disorders^33^. In the current study, C16, Ang-1, and the combination of C16 and Ang-1 alleviated PD-induced Syn reduction, indicating improved cognitive function in the drug-treated groups.

The expression of pS129-α-syn, a surrogate marker of PD progression, is related to the severity of motor impairment in PD patients^13^. A previous study showed that intracerebral injections of misfolded wild-type or mutant α-synuclein protein led to progressive neurodegeneration and α-synucleinopathy^13^. Both LRRK2 and α-synuclein are key factors in PD^11,13^ and are linked to microglial activation. Extracellular α-synuclein can directly initiate microglial activation and be phagocytosed by microglia, whereas LRRK2 is involved in the intrinsic regulation of microglial activation and autophagolysosomal degradation^11^. The injection of recombinant monomeric α-synuclein in the mouse SN was shown to induce nigral microglial activation at 24-h post-injection, and upregulate pro-inflammatory markers (i.e., IL-1β, IL6, TNFα, and COX-2) compared to control animals^30^. Gelders et al. investigated the role of infiltrating monocytes in α-synuclein-mediated neuroinflammation and neurodegeneration, and found that the infiltration of peripheral monocytes into the brain might play a crucial role in α-synuclein-mediated neuronal cell death^34^. In the current study, treatment with C16, Ang-1, or both suppressed peripheral monocyte infiltration in PD models, which was accompanied by the downregulation of pS129-α-syn.

Following inflammation in the CNS, astrocytes can proliferate to induce reactive astrogliosis, which can result in the formation of a glial scar and inhibit axonal regeneration. The current results revealed that C16 alone or in combination with Ang-1 reduced the proliferation of GFAP^+^ reactive astrocytes by improving the inflammatory microenvironment, thereby exerting a neuroprotective effect on PD animals.

The proteoglycan NG2 plays a fundamental role in proliferation, migration, and differentiation of pericytes and oligodendroglial precursor cells in the brain^12^. The BBB constitutes a neurovascular unit formed by microvascular endothelial cells, pericytes, and astrocytes. Brain pericytes are important regulators of BBB integrity and permeability. The loss of crosstalk among pericytes, endothelial cells, and astrocytes ultimately leads to BBB dysfunction^32^. Previous studies have shown that increased vessel leakage, pericyte apoptosis, and blood-retina-barrier breakdown are characteristic features of diabetic mouse retina^35^. In the current study, treatment with C16, Ang-1, or both increased the number of NG2^+^ pericytes in PD models. Ang-1 alone or in combination with C16 showed improved effects, likely due to their role in the promotion of pericyte-endothelium integrity and prevention of BBB dysregulation.

The dysregulation of the striatopallidal pathway is a key mechanism underlying the manifestation of PD symptoms. Opioid neuropeptides (dynorphin and enkephalin) are neurotransmitters that modulate synaptic plasticity and transmission, as well as striatal-based behaviors under both normal and pathological conditions, including PD^36^.

Dopaminergic projections from the substantia nigra terminate on striatal medium-sized spiny neurons containing GABA. The “direct” or “striatonigral” pathway is constituted by medium spiny neurons that express dopamine D1 receptors and the opioid peptide dynorphin, and directly project to the internal segment of the globus pallidus and the pars reticulata of the substantia nigra^37^. Dynorphin released from the terminals of the striatonigral and striatopallidal pathways acts as an endogenous modulator of glutamatergic transmission in the basal ganglia. In contrast, medium spiny neurons expressing opioid enkephalin and D2 receptors activate the “indirect” or “striatopallidal” pathway, which indirectly projects to basal ganglia output nuclei via the subthalamic nucleus and the external globus pallidus (or the globus pallidus in rodents)^38^. Dopamine normally inhibits transmission via the indirect pathway, whereas it facilitates transmission via the direct pathway^39,40^.

Our PD models showed decreased expression of prodynorphin and increased expression of preproenkephalin, suggesting the inhibition of the direct pathway and the induction of the indirect pathway. The downregulation of dynorphin has been reported in PD models^41^, and the expression of enkephalin can be induced by acute dopamine denervation in a similar model^41^. Therefore, changes in expression of both prodynorphin and preproenkephalin are related to abnormal behavior and pathological signs of PD, which may related to the protective effects of our drugs on medium spiny neurons^8^.

The anti-inflammatory effects of endogenous dynorphin protect against neurotoxin-induced nigrostriatal dopaminergic neuron damage^41^ through the inhibition of nicotinamide adenine dinucleotide phosphate (NADPH) oxidase-generated superoxide production in the microglia. Decreased superoxide production reduces the secretion of pro-inflammatory factors, including prostaglandins and cytokines, thereby dampening inflammation^41^.

c-fos is a neuronal activation marker that is upregulated in PD due to the disturbance of neural networks^42^. The combination treatment with C16 and Ang-1 further increases the expression of c-fos, suggesting further neuronal activation. In our animal models, the expression of DARPP-32 was not significantly different among groups, while phospho-DARPP-32 was upregulated compared to the control groups. The phosphorylation of DARPP-32 by cAMP-dependent protein kinase may contribute to behavioral changes related to dystonia^43^.

Microglial activation followed by reactive microgliosis exacerbates dopaminergic neuronal loss, fuels a self-renewing cycle, and thus facilitates the progression of PD^44^. Following MPTP insult, the number of microglia in the classical activation state (M1) is increased through inflammatory responses, while the number of M2 phenotype microglia, induced by anti-inflammatory cytokines and found to promote tissue remodeling and wound healing, is decreased^45^. Many endogenous noxious compounds that appear in the extracellular milieu following neuronal injury cause the activation of microglia and subsequent reactive microgliosis. Although MPTP/6-OHDA are known to damage dopaminergic neurons, the effects of these drugs on reactive microgliosis remain unclear^44^.

The combination of C16 and Ang-1 has also been shown to activate M2 phenotype and depress M1 phenotype microglia^27^. T helper type 2 cells reduce microglial activation and ameliorate neurodegenerative disorders via the activation of α_V_β_3_ integrins. The expressions of α_V_β_3_ integrins in Th2 cells are higher compared to Th1 cells, and the antibodies against α_V_ β_3_ integrins have been shown to diminish the inhibitory effects of Th2 cells on microglial activation^44,46^. As an agonist of α_V_β_3_ integrins, the C16 peptide also inhibits microglial activation and alleviates inflammation. Furthermore, the phosphoinositide 3-kinase (PI3K)/Akt axis is considered a promising target for the treatment of neuropathological disorders, such as PD^47^. The activation of the PI3K/Akt signaling pathway promotes tissue repair under neuro-inflammatory conditions^46^. Our previous study suggested that both C16 and Ang-1 activated the PI3K/Akt pathway to enhance cell viability and reduce apoptosis^27^.

However, making C16 plus Ang-1 an effective therapy in the clinical setting for inflammatory CNS diseases remains to be explored. Efforts are being made on dose adjustment and observing side effects, as well as investigation into the most effective form of the drug. The kinetics and pharmacodynamics of C16 still need to be addressed in preclinical studies. C16 is more easily dissolved in acidic solution, which may limit its medical use, while nano-sized vesicles may be a reliable solution for drug packaging.

In conclusion, MPTP and 6-OHDA were used to induced oxidative stress and chronic inflammation, and subsequent dopaminergic neuron degeneration, in rodents to model PD. The disturbance of the striatopallidal and striatonigral pathways resulted in abnormal behaviors and dystonia, accompanied by prodynorphin downregulation and preproenkephalin upregulation, suggesting the inhibition of the direct pathway and the activation of the indirect pathway. Tissue edema, blood vessel leakage, inflammatory cell infiltration, TH^+^/CHAT^+^ /GABA^+^ neuron death, and severe synaptophysin loss were observed in the striatum corpus and nigra substance of PD animals. Treatment with C16 or Ang-1 alone improved the inflammatory microenvironment, reduced the loss of dopaminergic neurons, improved cognitive and electrophysiological dysfunction, and attenuated functional disability in model animals. The combined treatment with both C16 and Ang-1 showed a synergistic effect on the amelioration of Parkinsonism.

## Methods

### Animals

Male C57/BL6 mice (20–25 g) and male Sprague-Dawley rats (200–250 g) were purchased from Vital River Lab Animal Technology (Beijing, China) and housed in an environment with 40–60% humidity, a temperature of 22 ± 1 °C, and a 12 h light/dark cycle. All animals access to water and food *ad libitum*. This study was approved by the Animal Care Committee of the Chinese Academy of Medical Sciences and performed in accordance with the NIH guidelines.

### Establishment of the mouse model of PD

Seventy-five mice were randomly divided to five groups (*n* = 15 per group): C16, Ang-1, C16 + Ang-1, vehicle, and normal control. To establish the PD model, mice in the C16, Ang-1, C16 + Ang-1, and vehicle groups were injected intraperitoneally (i.p.) with 1-methyl-4-phenyl-1,2,3,6-tetrahydropyridine (MPTP, dissolved in 0.9% saline; Sigma-Aldrich, St Louis, MO, USA) at a dose of 30 mg/kg for five consecutive days, while the normal control group was injected i.p. with an equal volume of normal saline^7^. All drug treatments began one day after the completion of the MPTP injections. Mice in the drug-treated groups were injected intravenously with a 1 mL solution containing C16 (2 mg/100 g) and/or Ang-1 (400 µg/100 g) every day for 2 weeks. The vehicle and normal control groups were injected intravenously with 1 mL of distilled phosphate buffer saline (PBS) via the tail vein^48^.

### Establishment of the rat model of PD

Seventy-five rats were randomly assigned to five groups (*n* = 15 per group): C16, Ang-1, C16 + Ang-1, vehicle, and normal control. Except for the normal control group, rats were anesthetized with sodium pentobarbital via i.p. injection, then fixed in a stereotaxic apparatus (David Kopf Instruments, Tujunga, CA, USA) and the skull was exposed. A burr hole (1 mm) was drilled into the cranial cavity according to the following coordinates: mediolateral, 2.7 mm from the midline; anteroposterior, 0.8 mm from the bregma; dorsoventral, −5.2 and −4.5 mm, respectively, from the skull. Subsequently, 6-hydroxydopamine (6-OHDA; 20 µg dissolved in 4 µL normal saline supplemented with 0.01% (w/v) ascorbic acid) was infused into the right striatum of the rat, 2 µL at each coordinate, at a constant rate of 0.2 µL/min using a 10-µL Hamilton syringe. At the end of the infusion, the syringe was left in place for an additional 5 min before being slowly retracted. All rats were kept warm using a heating pad during the surgery and recovery period.

Animals in the drug-treated groups were intravenously injected with a 1 mL solution containing C16 (2 mg/100 g) and/or Ang-1 (400 µg/100 g) every day for 2 weeks. Animals in the vehicle and normal control groups were intravenously administered with 1 mL of distilled PBS via the tail vein. All drug treatments began one day after the 6-OHDA injection.

### Open field test

The open field test was used to detect locomotor activity dysfunction. It began two days after the 6-OHDA infusion/last MPTP injection and lasted for 7 days. Mice/rats received adaptive training three times before PD induction. Mice/rats were placed individually in an open arena (32 × 44 × 44 cm^3^). A video camera was placed over the arena to detect the position of the animal. The activity of the animal was recorded for 5 min under weak light illumination (40 W). The mean velocity, activity, and total distance traveled were collected and analyzed using the EthoVision video tracking system (Noldus, Wageningen, Netherlands). The average values of three independent tests from the same animal were used for statistical analysis.

### Muscular coordination test

The rotarod test was performed once a day for 8 days to assess the motor-coordination ability of the rodents. It began 2 days after the 6-OHDA infusion/last MPTP injection. Animals were trained three times before PD induction. Each animal was placed on a rotarod (8 × 9 cm; KN-75, Natsume Seisakusho) that rotated at 15 rpm in the clockwise direction. The time that the test animal stayed on the rotarod until falling was recorded^49^.

### Novel object recognition (NOR) test

This test evaluated the ability to recognize a novel object in an environment by comparing the amount of time exploring a novel and a familiar object. It is used to assess declarative memory (i.e., memory of events and facts) in rodents. In a soundproof room under low brightness conditions, the test animal was placed in a rectangular chamber (40 × 50 × 60 cm) with a glass front side. There were three phases of the test: habituation (day 1), familiarization (day 2), and the test phase (day 3). In the first phase (habituation), a test animal could freely explore the entire chamber for 20 min and was then put back to its cage. In the second phase (familiarization), the animal was placed in a chamber containing two identical sample objects (A + A) and allowed to explore for 5 min. Ninety minutes later, the animal was placed in a chamber with two different sample objects (A + B) and allowed to explore for 5 min. The next day, the animal was placed in the chamber with two objects. One was identical to the original sample object (A) and the other was a novel object (C). The animal was allowed to freely explore for 5 min, during which long-term memory was evaluated. The novelty preference was defined as the difference in time that each animal spent to locate the novel object (N) and the familiar object (F). The discrimination score was calculated as (N–F)/(N + F) in seconds^50^.

### Electrophysiological analysis

Electrophysiological experiments were performed using a telemetry-based two lead EMG transmitter (F20-EET, Data Sciences International, St. Paul, MN, USA). One week following PD induction, animals were anesthetized with a ketamine cocktail. The wire electrodes were tunneled to both hindlimbs subcutaneously by separating the skin from the muscle using blunt dissection. To allow electrodes to be inserted through the quadriceps and biceps femoris muscles, a small incision was made over the thigh. Sutures were used to hold the electrodes in place. EMG data were acquired using DataQuest Acquisition hardware (Data Sciences International) and analyzed using LabChart (ADInstuments, Colorado Springs, CO, USA). The transmitter and the receiver communicated at a frequency of 455 kHz, which reduced signal contamination from surrounding electronics^51^.

### Perfusion and tissue processing

All groups of animals (*n* = 5 per group) were sacrificed at four weeks post MPTP or 6-OHDA injection. They were anesthetized with sodium pentobarbital and intracardially perfused with cold saline followed by 4% paraformaldehyde (pH = 7.4, diluted in 0.1 M phosphate buffer) before brain dissection. The brain tissues were fixed in 4% paraformaldehyde for 4 h and immersed in 30% sucrose diluted in PBS until the tissue sank to the bottom of the container. A Leica cryostat and a freezing microtome (Buffalo Grove, IL, USA) were used to cut tissues into 20-μm coronal sections. Finally, the sections were mounted for histopathological and immunofluorescence analyses.

### Transmission electron microscopy

A portion of the striatum and substantia nigra tissues were fixed in a 2.5% glutaraldehyde solution, then immersed in 1% osmium tetroxide overnight at 4 °C. After being washed three times with PBS, the sections were examined by transmission electron microscope (TEM; JEOL, Tokyo, Japan)^48^.

### Evans Blue staining

Evans Blue (EB) dye extravasation into the brain indicates tissue edema and compromised blood vessel integrity. The vascular permeability of the BBB (*n* = 5 per group) was determined by modified EB staining. Briefly, mice and rats at 4 weeks P.I. were anesthetized with sodium pentobarbital (60 mg/kg) via i.p. injection. Animals were then infused with 2% EB dye (4 mL/kg, dissolved in 0.9% normal saline) via the right femoral vein for 5 min at 37 °C. After 2 h, blood vessels were perfused with 300 mL of normal saline to wash out residual dye. EB-stained sections were then visualized under an ultraviolet light filter with red laser excitation. Red staining indicates high vascular permeability in the tissue. The staining intensity was determined using Image J software (NIH, Bethesda, MD, USA). Half of the tissue samples were isolated, homogenized in 750 μL of *N, N-*dimethylformamide (Sigma-Aldrich), and maintained in the dark at room temperature for 72 h. Then, the suspension was centrifuged at 10000× *g* for 25 min before spectrophotometer reading at 610 nm (Molecular Devices OptiMax, USA). The dye concentration (μg/g tissue weight) was calculated from a standard curve^48^.

### Immunofluorescence staining

A ring of wax was applied around tissue sections using a water-repellent PAP pen (Invitrogen, Carlsbad, CA, USA) as previously described^50^. The sections were then rinsed with 0.01 M Tris-buffered saline for 10 min, followed by permeabilization and blocking with 0.3% Triton X-100/10% normal goat serum for half an hour, and incubation with the following antibodies at 4 °C overnight: rabbit/mouse anti-synaptophysin (Syn, ab32127, 1:500; Abcam, Cambridge, MA, USA), anti-prodynorphin (PA5-96439,1:500; Thermo Fisher Scientific, Waltham, MA, USA), anti-preproencephalin (sc-47705, 1:200; Santa Cruz Biotechnology), anti-tyrosine hydroxylase (TH, ab137869, 1:500; Abcam), anti-choline acetyltransferase (CHAT, ab181023, 1:500; Abcam), anti-c-fos (ab208942, 1:200; Abcam), anti-phospho-DARPP-32 (Thr34, PA5-39693, 1:500; Thermo Fisher Scientific), anti-DARPP-32 (PA5-85788, 1:500; Thermo Fisher Scientific), anti-GFAP (sc-166481, 1:200; Santa Cruz Biotechnology), CD-3 (sc-20047, 1:200; Santa Cruz Biotechnology), LRRK2 (sc-130159, 1:200; Santa Cruz Biotechnology), anti-ZO-1 (sc-33725, 1:200; Santa Cruz Biotechnology), anti-LC3β (sc-376404, 1:200; Santa Cruz Biotechnology), anti-caspase 3 (sc-56053, 1:200; Santa Cruz Biotechnology), Iba-1 (sc-32725, 1:200; Santa Cruz Biotechnology), and NG2 (sc-53389, 1:200; Santa Cruz Biotechnology), DA (ab6427, 1:500; Abcam), GABA-transporter (ab177483, 1:500; Abcam) and anti-pS129-alpha-syn (ab51253, 1:500; Abcam). After rinsing with PBS three times, the sections were stained with a FITC/TRIFC-conjugated goat anti-rabbit/mouse IgG secondary antibody (sc-2010, sc-2012, sc-2780, 1:200; Santa Cruz Biotechnology) at 37 °C for 60 min and mounted with Gel Mount antifade aqueous mounting medium (Southern Biotech). Inactive antibody controls were used to confirm the specificity of IHC labeling. Five sections (three visual fields per slide) of the anterior horns and motor cortex were randomly selected for counting. Images were captured at 200× magnification using a light microscope (Buffalo Grove) and analyzed using Image J software (Bethesda, MD, USA).

### Quantification of inflammatory cell infiltration

Tissue sections stained with the pan-leukocyte marker CD-3 were observed under a light microscope by an experienced pathologist blinded to the experiment. Five randomly selected fields of view per section were examined. The severity of inflammatory cell infiltration was scored as follows^52^: 0, no inflammation; 1, cellular infiltrates only detected around meninges and blood vessels; 2, mild infiltrates detected in parenchymal tissues (1–10 inflammatory cells per slide); 3, moderate infiltrates observed in parenchymal tissues (11–100 inflammatory cells per slide); and 4, severe infiltrates in parenchymal tissues (>100 inflammatory cells per slide).

### Stereological analysis

Tissue samples were immunostained for the DA neuron marker (TH), astrocyte marker (GFAP), microglia marker (Iba-1), and medium spiny neuron marker (GABA transporter). The number of positively stained cells were quantified according to the optical fractionator method using the Olympus Computer Assisted Stereological Toolbox (CAST)-Grid system (Olympus, Denmark). The system consists of an Olympus BH-2 microscope with an X–Y motor stage controlled by the CAST-Grid software (version 1.10) and a microcator (Heidenhain, ND 281) connected to the stage to indicate the distance in the Z direction. Stereo Investigator was performed by an observer blinded to the experiment. The stage-controlling computer systematically moved until the entire delineated field was sampled. The positive cell bodies of mice were analyzed from three sections at the medial region of the SNpc, around the medial terminal nucleus (approximately from −3.08 to −3.28 mm from the bregma), at regular predetermined intervals (x = 100 μm, y = 80 μm, counting frame 60 × 60 μm). The positive cell bodies of rats were analyzed from nine sections spanning the entire SNpc (approximately AP −4.5 to −6.0 relative to the bregma) at predetermined regular intervals (x = 125 μm, y = 125 μm, counting frame 80 × 80 μm). In both models, the region of interest (ROI) was first defined using the ×4 objective and cells were counted using a ×100 oil immersion objective (Olympus BX51, Olympus, Tokyo, Japan). In the analysis of mouse tissue samples, every third section was selected with the striatum between +0.26 mm and +0.98 mm from the bregma. In the analysis of rat tissue samples, the striatum was quantified every 10th section (with a distance of 400 μm) located between 1.7 mm anterior and 1.4 mm posterior to the bregma (∼8 sections). The medial border of the striatum was defined by the lateral ventricle and the dorsal and lateral borders by the corpus callosum. Stereo Investigator was used to outline the region at ×4 magnification and then cells stained with anti-TH, anti-GABA transporter, anti-GFAP, or anti-Iba-1 were counted using a ×100 oil immersion objective.

### Enzyme-linked immunosorbent assay (ELISA)

Animals were sacrificed at 3- and 8-week P.I. by decapitation (*n* = 5 per group per time point). Peripheral blood samples were collected at 4 °C using heparin as an anticoagulant and then immediately centrifuged at 1000 × *g* for 20 min followed by 10000 × *g* for 10 min at 4 °C before storage at −80 °C. To measure the serum levels of cytokines, blood samples were added to 96-well plates pre-coated with anti-IL-6(mouse, M6000B; rat, R6000B, R&D Systems, Minneapolis, MN, USA), anti-ROS (mouse, MBS2601061, MyBioSource, San Dieg, USA; Rat, RJ15780, Shanghai Renjie Biological Technology Co.,Ltd, Shanghai, China) and anti-γ-aminobutyric acid primary antibodies (GABA, mouse, MBS723819; rat, MBS269152, MyBioSource, San Dieg, USA). After 1-h incubation at 37 °C, samples were incubated with a horse radish peroxidase (HRP)-conjugated goat anti-rabbit IgG secondary antibody (ab8226, 1:1000; AbCam, MA) for 60 min at 37 °C. The optical density of the protein was measured using a microplate reader (Model 680; Bio-Rad) at 450 nm and the results were analyzed using the GraphPad Prism software (version 4; GraphPad Prism Software, CA, USA).

### Western blot

Mice/rats were sacrificed at 2- and 8-week P.I. by decapitation (*n* = 5 per group per time point). A 10-mm lumbar SC segment and the entire brain cortex were isolated from each animal. Protein samples were separated by 12% SDS-PAGE electrophoresis and transferred onto polyvinylidene difluoride membranes. The membranes were then incubated with the following primary antibodies at room temperature for 12 h: rabbit/mouse anti-synaptophysin (ab32127, 1:500; Abcam), anti-prodynorphin (PA5-96439, 1:1000; Thermo Fisher Scientific) and anti-preproencephalin (sc-47705, 1:500; Santa Cruz Biotechnology), anti-TH (ab137869, 1:1500; Abcam), anti-CHAT (ab181023, 1:1500; Abcam), anti-c-fos (ab208942, 1:1000; Abcam), anti-phospho-DARPP-32 (Thr34, PA5-39693, 1:1000; Thermo Fisher Scientific), anti-DARPP-32 (PA5-85788, 1:1000; Thermo Fisher Scientific), anti-GFAP (sc-166481, 1:500; Santa Cruz Biotechnology), CD-3 (sc-20047, 1:500; Santa Cruz Biotechnology), LRRK2 (sc-130159, 1:500; Santa Cruz Biotechnology), anti-ZO-1 (sc-33725, 1:500; Santa Cruz Biotechnology), anti-LC3β (sc-376404, 1:500; Santa Cruz Biotechnology), anti-caspase 3 (sc-56053, 1:500; Santa Cruz Biotechnology). DA (ab6427, 1:1500; Abcam), GABA-transporter (ab177483,1:1500; Abcam), NF-κB (ab16502,1:1500; Abcam), COX-2 (ab179800, 1:1500; Abcam) and anti-pS129-α-syn (ab51253, 1:1000; Abcam). To normalize protein bands to the loading control, membranes were washed with TBST and re-probed with rabbit anti-β-actin antibody (ab8226, 1:5000; Abcam) followed by incubation with a peroxidase-conjugated goat anti-rabbit antibody (1:5000; Santa Cruz Biotechnology) and ECL detection. For the negative control, the primary antibody was omitted. All blots derived from the same experiment and were processed in parallel.

### Statistical analysis

Differences between protein levels were analyzed with two-way analysis of variance (ANOVA), followed by Tukey’s post-hoc test. The Kruskal–Wallis nonparametric one-way ANOVA was used for the analysis of data presented as percentages. Differences between histological scores were analyzed using the Mann-Whitney *U* test. Continuous data were presented as mean ± standard deviation (SD). All data were analyzed using SPSS 13.0 software, and *p*-values less than 0.05 were considered statistically significant. All statistical graphs were created with GraphPad Prism Version 4.0 (GraphPad Prism Software, Inc., San Diego, CA).

### Reporting summary

Further information on research design is available in the Nature Research Reporting Summary linked to this article.

## Supplementary information

Supplementary Information

Reporting Summary

## Data Availability

The data that support the findings of this study are available from the corresponding author upon reasonable request.
