# Supplementary material for: Adjusting vascular permeability, leukocyte infiltration, and microglial cell activation to rescue dopaminergic neurons in rodent models of Parkinson’s disease
Source: NPJ Parkinsons Dis. 2021 Oct 8;7:91. doi: 10.1038/s41531-021-00233-3 (PMC8501121; doi:10.1038/s41531-021-00233-3)
Supplement: Supplementary file 1 — Supplementary Information [file 41531_2021_233_MOESM1_ESM.pdf]

Adjusting vascular permeability, leukocyte infiltration, and microglial cell activation to rescue dopaminergic neurons in rodent models of Parkinson's disease

Running title: C16 plus Ang-1 suppresses inflammation in PD model

Hua-Ying Cai[1], Xiao-Xiao Fu[2#*], Hong Jiang[3], Shu Han[2#*]

[1]Department of Neurology, Sir Run Run Shaw Hospital, Medical College, Zhejiang University, Hangzhou, China

[2]Institute of Anatomy and Cell Biology, Medical College, Zhejiang University, Hangzhou, China

[3]Department of Electrophysiology, Sir Run Run Shaw Hospital, Medical College, Zhejiang University, Hangzhou, China

[#]The authors have made an equal contribution to the article

***Corresponding author:** Shu Han

Institute of Anatomy and Cell Biology, Medical College, Zhejiang University, 866 Yuhangtang Road, 310058, Hangzhou, China.

Tel: 86-571-88208160; Fax: 86-571-88208094; E-mail: Han00shu@zju.edu.cn

***Co-corresponding author:** Xiao-Xiao Fu

Institute of Anatomy and Cell Biology, Medical College, Zhejiang University, 866 Yuhangtang Road, 310058, Hangzhou, China.

Tel: 86-571-88208160; Fax: 86-571-88208094; E-mail: 21818569@zju.edu.cn

Supplementary materials

Supplementary Figure legends

Supplementary Figure 1. (a-d) Rats subjected to systemic injection of 6-OHDA (c, d) showed synchronously contraction of agonist (quadriceps femoris) and antagonist (bicep femoris) only in the left side of the lower limbs (6-OHDA was injected via the right striatum), as measured by the amplitude of wave (uV) when compared to the normal control group (a, b). The antagonist muscle of the model animals treated with C16 alone (e, f), Ang-1 alone (g, h), and especially the combination of C16 and Ang-1 (i, j) did not contract with the agonist muscles synchronously after stimulation. Abbreviations: 6-OHDA, 6-hydroxydopamine; C16, peptide (KAFDITYVRLKF) that can selectively bind integrin $\alpha_v\beta_3$; Ang-1, angiopoietin-1.

Supplementary Figure 2. (a-j): The immunostaining of LRRK2$^+$ cells (green) in the striatum showed that the number of LRRK2$^+$ cells was increased in vehicle-treated PD models (b, g) compared to the normal control group (a, f). C16 (c, h), Ang-1 (d, i), and especially C16 plus Ang-1 (e, j) downregulated the expression of LRRK2 in PD models. Scale bar = 100 µm. (k): Quantification of LRRK2$^+$ cells. (l, m) Western blot analysis showed that the expression of LRRK2 was elevated in the vehicle-treated group (l, mice; m, rats), but reduced by C16, Ang-1, and the combination of C16 and Ang-1. (o, p): Quantification of western blot (o, mice; p, rats). (a), $p < 0.05$ versus the normal control group; (b), $p < 0.05$ versus the vehicle group (c), $p < 0.05$ versus the C16-treated group (d), $p < 0.05$ versus the Ang-1-treated group. LRRK2 protein:286 kDa; β-actin protein: 42 kDa; Abbreviations: LRRK2, leucine-rich-repeat kinase 2;

C16, peptide (KAFDITYVRLKF) that can selectively bind integrin $\alpha_v\beta_3$; Ang-1, angiopoietin-1.

Supplementary Figure 3. Quantification of the western blot showed that the expression of pro-inflammatory factors, NF-κB (a, mice; b, rats) and COX-2 (c,mice; d, rats), and pS129-α-syn (e, mice; f, rats) were upregulated, while the expression of DA (g, mice; h, rats) and GABA (i, mice; j, rats) were decreased in vehicle-treated PD models compared to the normal controls. Ang-1, C16, and C16 plus Ang-1 reversed the aberrant expression of these proteins in PD animals. (a), $p < 0.05$ versus the normal control group; (b), $p < 0.05$ versus the vehicle group (c), $p < 0.05$ versus the C16-treated group (d), $p < 0.05$ versus the Ang-1-treated group. NFKB protein:60 kDa; COX2 protein:69 kDa; PS129-a-SYN protein:14 kDa; Dopamine protein: 68 kDa; GABA protein: 67 kDa; β-actin protein molecular weight: 42 kDa; Abbreviations: NF-κB, nuclear factor-kappa B; COX-2, cyclooxygenase 2; DA, dopamine; GABA, gamma-aminobutyric acid; PD, Parkinson's disease; C16, peptide (KAFDITYVRLKF) that can selectively bind integrin $\alpha_v\beta_3$; Ang-1, angiopoietin-1.

Supplementary Figure 4. The CNS of the vehicle-treated group exhibited BBB damage, vascular leakage (a-e, rats; k-o, mice), and destruction of tight junctions (f-j, rats; p-t, mice). Severe vasculature leakage was observed in vehicle-treated mice and rats (b, l). The expression of ZO-1, a tight junction protein expressed between endothelial cells lining blood vessels, was downregulated (g, q) compared to the normal controls (a, f, k, p). However, PD animals treated with C16 (c, h, m, r), Ang-1 (d, i, n, s), and especially the combination of C16

plus Ang-1 (e, j, o, t) showed greatly reduced leakage from blood vessels (u). The expression of ZO-1 was also increased in PD mice/rats treated with C16, Ang-1, and especially C16 plus Ang-1 (v). (a), $p < 0.05$ versus the normal control group; (b), $p < 0.05$ versus the vehicle group (c), $p < 0.05$ versus the C16-treated group (d), $p < 0.05$ versus the Ang-1-treated group. Abbreviations: CNS, central nervous system; BBB, blood-brain barrier; ZO-1, zonula occludens-1; PD, Parkinson's disease; C16, peptide (KAFDITYVRLKF) that can selectively bind integrin $\alpha_v\beta_3$; Ang-1, angiopoietin-1.

Supplementary Figure 5. Quantification of CHAT immunostaining revealed a notable reduction in the number of $CHAT^+$ neurons in the corpus striatum and substantia nigra of PD models (a-o, rats; p-y, mice). C16, Ang-1, and especially the C16 plus Ang-1 treatment markedly induced CHAT expression in both models (i, ii). (a), $p < 0.05$ versus the normal control group; (b), $p < 0.05$ versus the vehicle group (c), $p < 0.05$ versus the C16-treated group (d), $p < 0.05$ versus the Ang-1-treated group. Scale bar = 100 μm. Abbreviations: CHAT, choline acetyltransferase; C16, peptide (KAFDITYVRLKF) that can selectively bind integrin $\alpha_v\beta_3$; Ang-1, angiopoietin-1.

Supplementary Figure 6. Caspase-3-positive neurons were detected by immunofluorescence staining. The PD model groups showed more caspase-3-positive cells in the corpus striatum and nigra substance (a-j: rats; k-t: mice). Treatment with C16, Ang-1, and especially C16 plus Ang-1 remarkably decreased the number of caspase-3-positive neurons in both models (u, v). (a), $p < 0.05$ versus the normal control group; (b), $p < 0.05$ versus the vehicle group (c), $p <$

0.05 versus the C16-treated group (d), $p < 0.05$ versus the Ang-1-treated group. Scale bar = 100 μm. Abbreviations: PD, Parkinson's disease; C16, peptide (KAFDITYVRLKF) that can selectively bind integrin $\alpha_v\beta_3$; Ang-1, angiopoietin-1.

Supplementary Figure 7. Quantification of LC3 immunostaining showed an increase in the number of $LC3^+$ cells in the corpus striatum and nigra substance of PD animals (a-j, rats; k-o, mice). C16, Ang-1, and especially C16 plus Ang-1 significantly suppressed LC3 expression in both models (p). (a), $p < 0.05$ versus the normal control group; (b), $p < 0.05$ versus the vehicle group (c), $p < 0.05$ versus the C16-treated group (d), $p < 0.05$ versus the Ang-1-treated group. Scale bar = 100 μm. Abbreviations: LC3β: microtubule-associated protein light chain 3β; C16, peptide (KAFDITYVRLKF) that can selectively bind integrin $\alpha_v\beta_3$; Ang-1, angiopoietin-1.

Supplementary Figure 8. Quantification of cFOS in PD models (l, mice; m, rats) revealed that treatment with C16, Ang-1, and especially C16 plus Ang-1 increased the expression of cFos. (a), $p < 0.05$ versus the normal control group; (b), $p < 0.05$ versus the vehicle group (c), $p < 0.05$ versus the C16-treated group (d), $p < 0.05$ versus the Ang-1-treated group. Scale bar = 100 μm. Abbreviations: C16, peptide (KAFDITYVRLKF) that can selectively bind integrin $\alpha_v\beta_3$; Ang-1, angiopoietin-1; PD, Parkinson's disease.

Supplementary Figure 9. Quantification of immunostaining showed that the number of $pDARPP\text{-}32^+$ cells in the vehicle-treated group was notably increased compared to the normal controls, while C16, Ang-1, and especially C16 plus Ang-1 treatment evidently inhibited the

upregulation of pDARPP-32. (a), $p < 0.05$ versus the normal control group; (b), $p < 0.05$ versus the vehicle group (c), $p < 0.05$ versus the C16-treated group (d), $p < 0.05$ versus the Ang-1-treated group. Scale bar = 100 μm. Abbreviations: DARPP, dopamine- and cAMP-regulated neuronal phosphoprotein; pDARPP-32, phosphorylated DARPP-32; C16, peptide (KAFDITYVRLKF) that can selectively bind integrin $\alpha_v\beta_3$; Ang-1, angiopoietin-1.

Supplementary Figure 10. Quantification of immunostaining showed that the number of the pdyn+ cells in the vehicle-treated group was notably decreased compared to the normal controls, while C16, Ang-1, and especially C+A treatment significantly upregulated pdyn in both models (u, v). (a), $p < 0.05$ versus the normal control group; (b), $p < 0.05$ versus the vehicle group (c), $p < 0.05$ versus the C16-treated group (d), $p < 0.05$ versus the Ang-1-treated group. Scale bar = 100 μm. Abbreviations: pdyn, prodynorphin; C16, peptide (KAFDITYVRLKF) that can selectively bind integrin $\alpha_v\beta_3$; Ang-1, angiopoietin-1.

Supplementary Figure 11. Quantification of PPE+ cells. The immunostaining results showed that the number of PPE+ cells in the nigra substance was greatly increased in PD models (a-f, rats; g-k, mice). C16, Ang-1, and the C+A treatment markedly decreased the expression of PPE in both models (l, m). (a), $p < 0.05$ versus the normal control group; (b), $p < 0.05$ versus the vehicle group (c), $p < 0.05$ versus the C16-treated group (d), $p < 0.05$ versus the Ang-1-treated group. Scale bar = 100 μm. Abbreviations: PPE, preproenkephalin; C16, peptide (KAFDITYVRLKF) that can selectively bind integrin $\alpha_v\beta_3$; Ang-1, angiopoietin-1.

Supplementary Figure 12. The original unretouched blots presented in Figure 5. All of the Loading controls were run on the same blot gel as the sample bands.

Supplementary Figure 13. The original unretouched blots presented in Figure 12. All of the Loading controls were run on the same blot gel as the sample bands.

Supplementary Figure 14. The original unretouched blots presented in Figure 15. All of the Loading controls were run on the same blot gel as the sample bands.

Supplementary Figure 15. The original unretouched blots presented in Supplementary Figure 2. All of the Loading controls were run on the same blot gel as the sample bands.

Supplementary Figure 16. The original unretouched blots presented in Supplementary Figure 3. All of the Loading controls were run on the same blot gel as the sample bands.

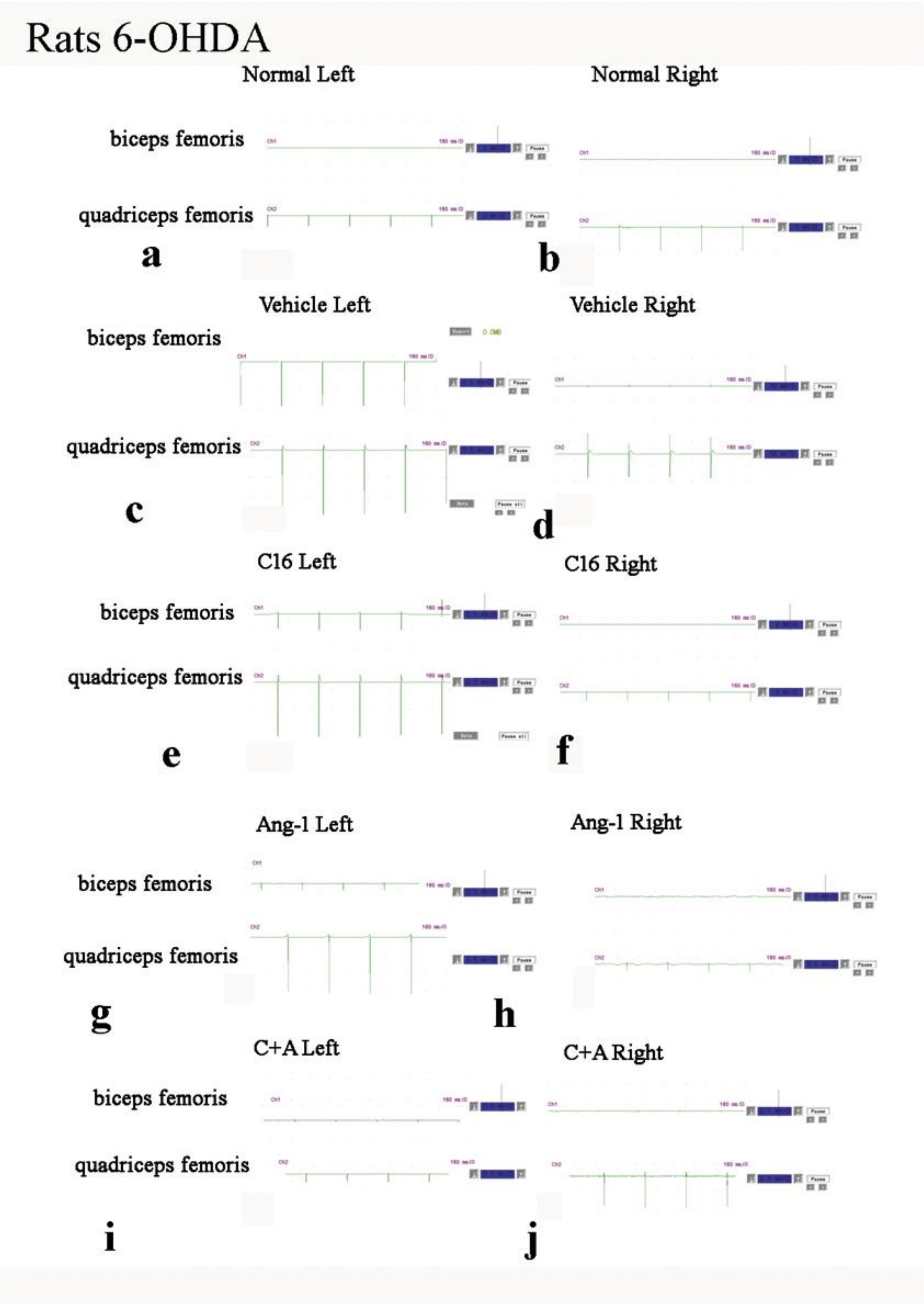

Supplementary Figure 1

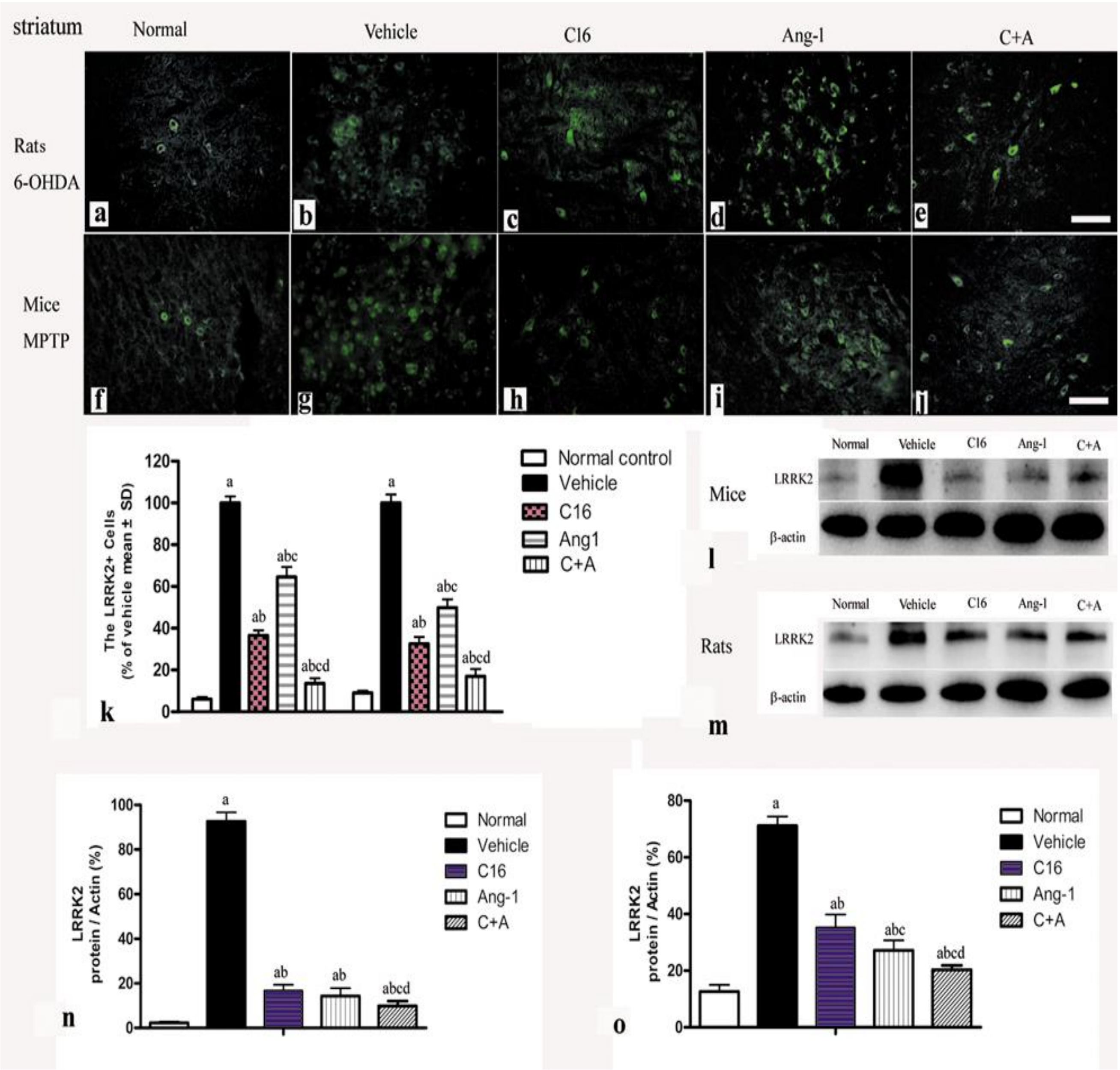

Supplementary Figure 2

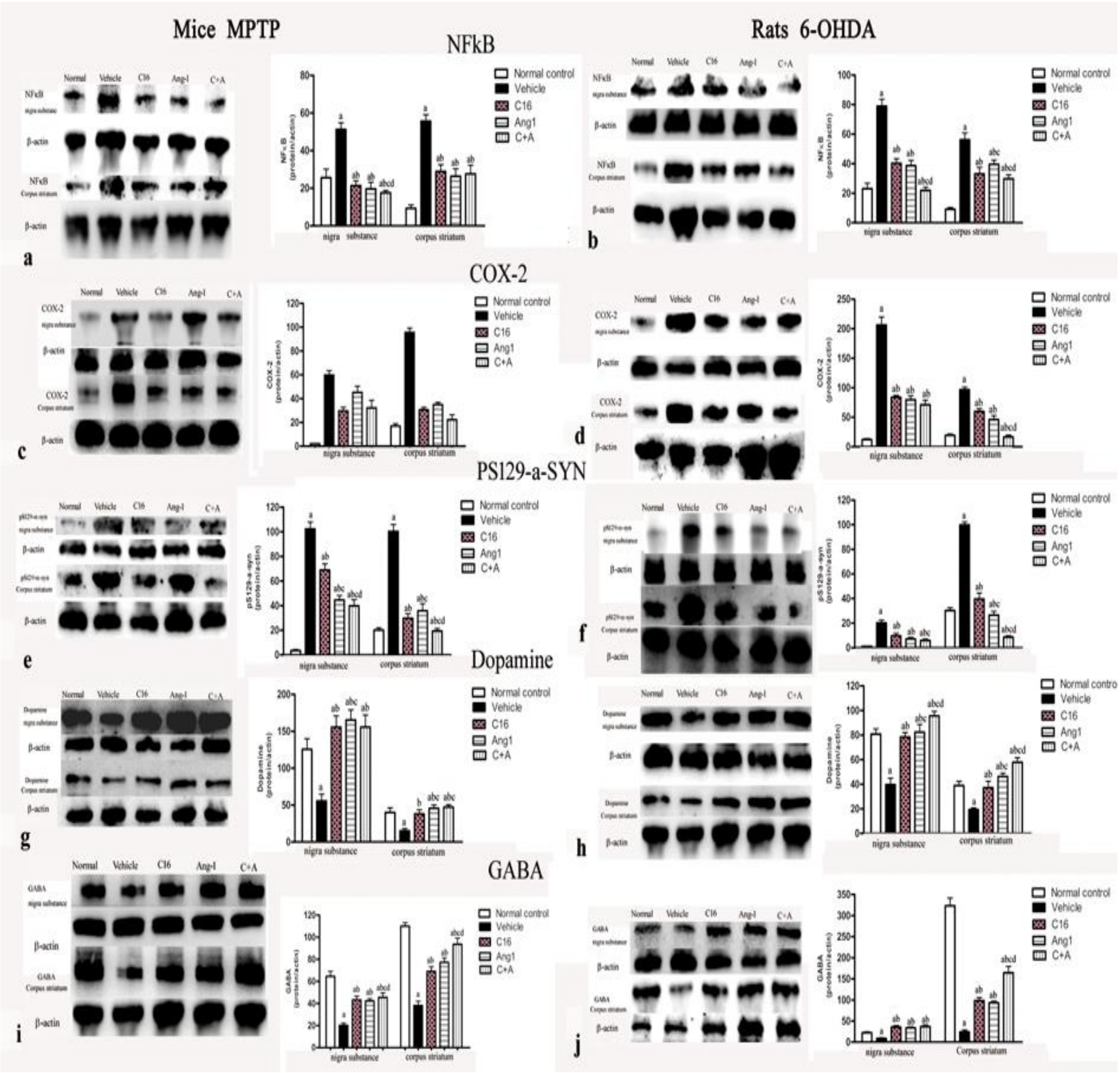

Supplementary Figure 3

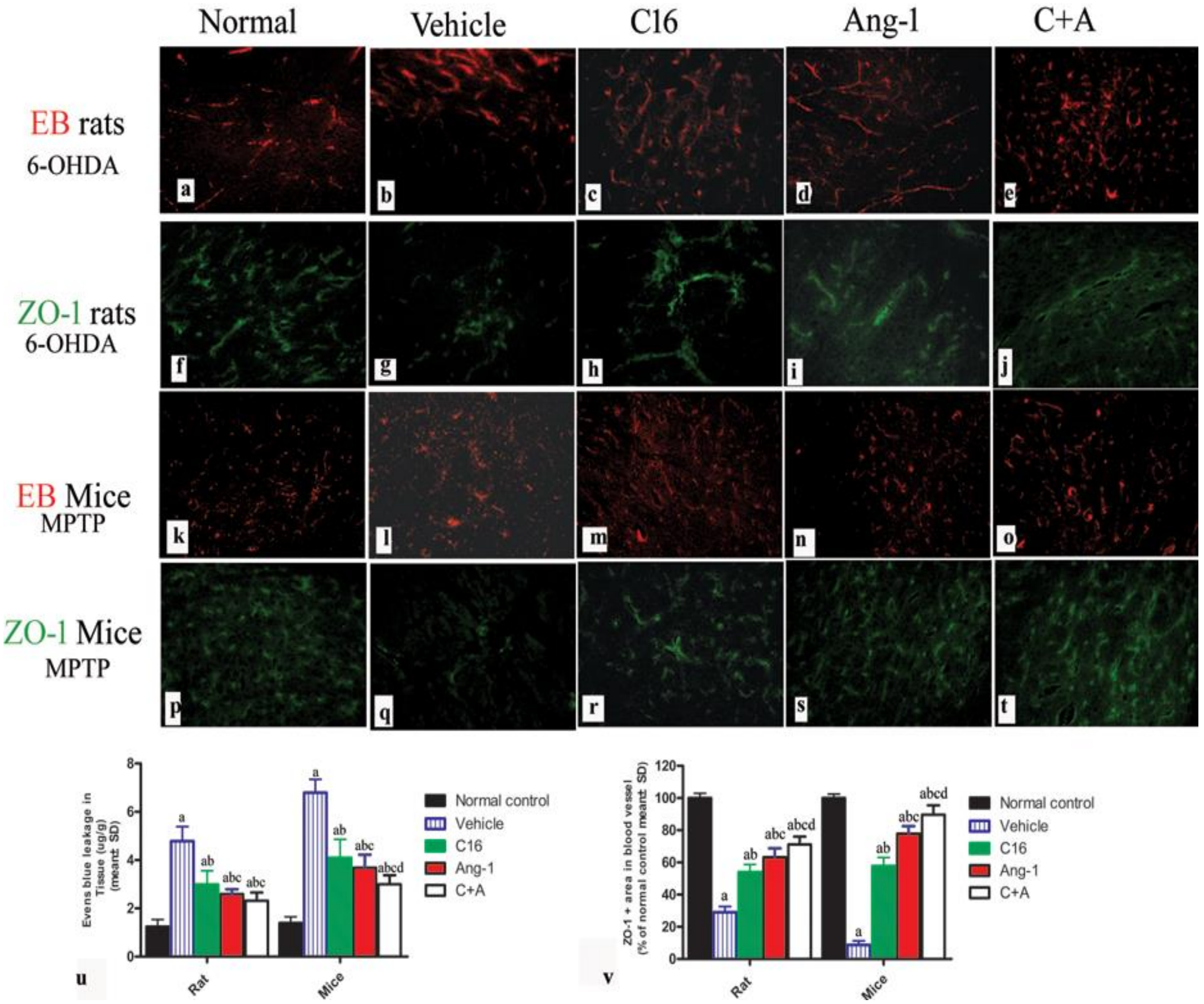

Supplementary Figure 4

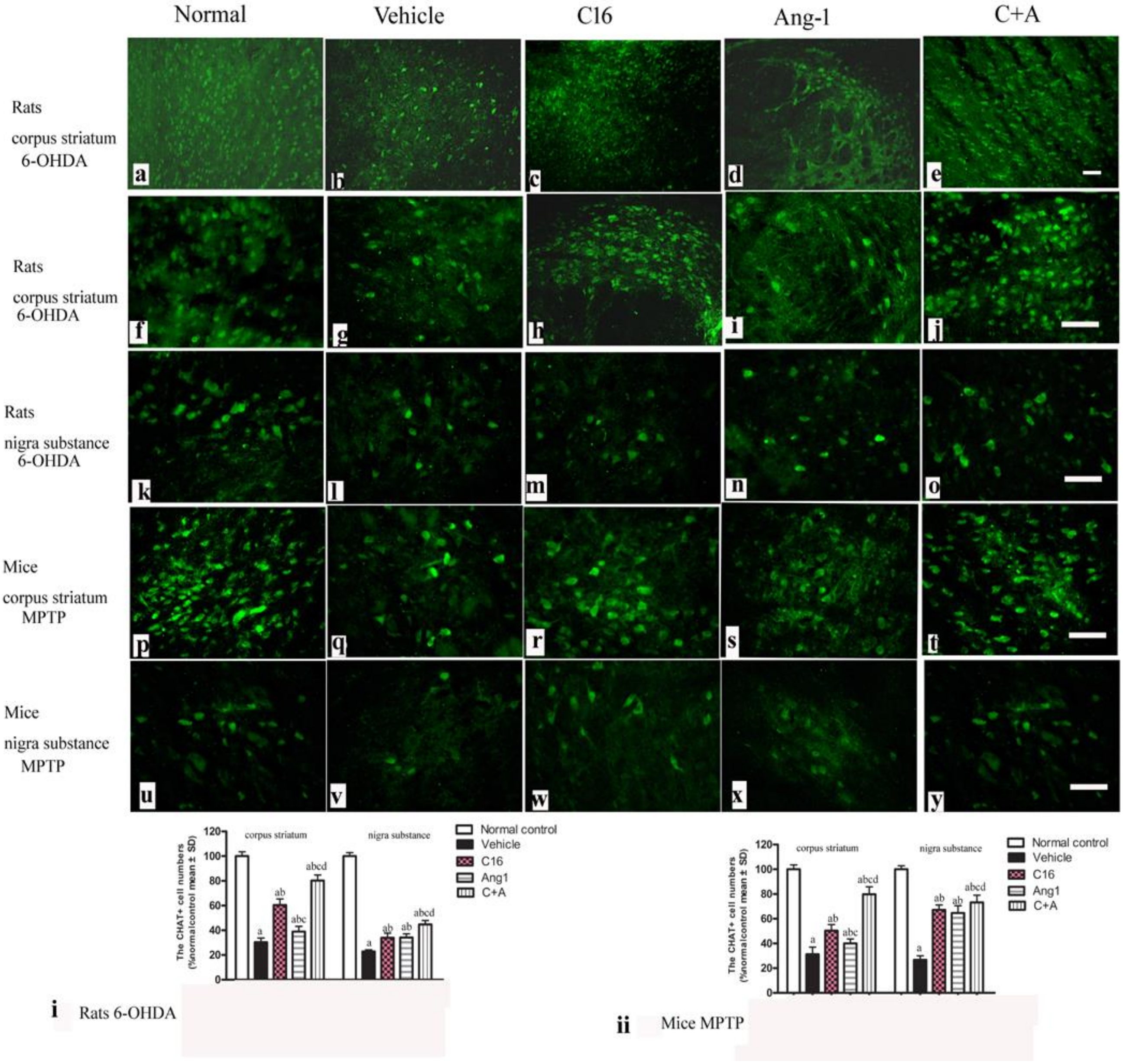

Supplementary Figure 5

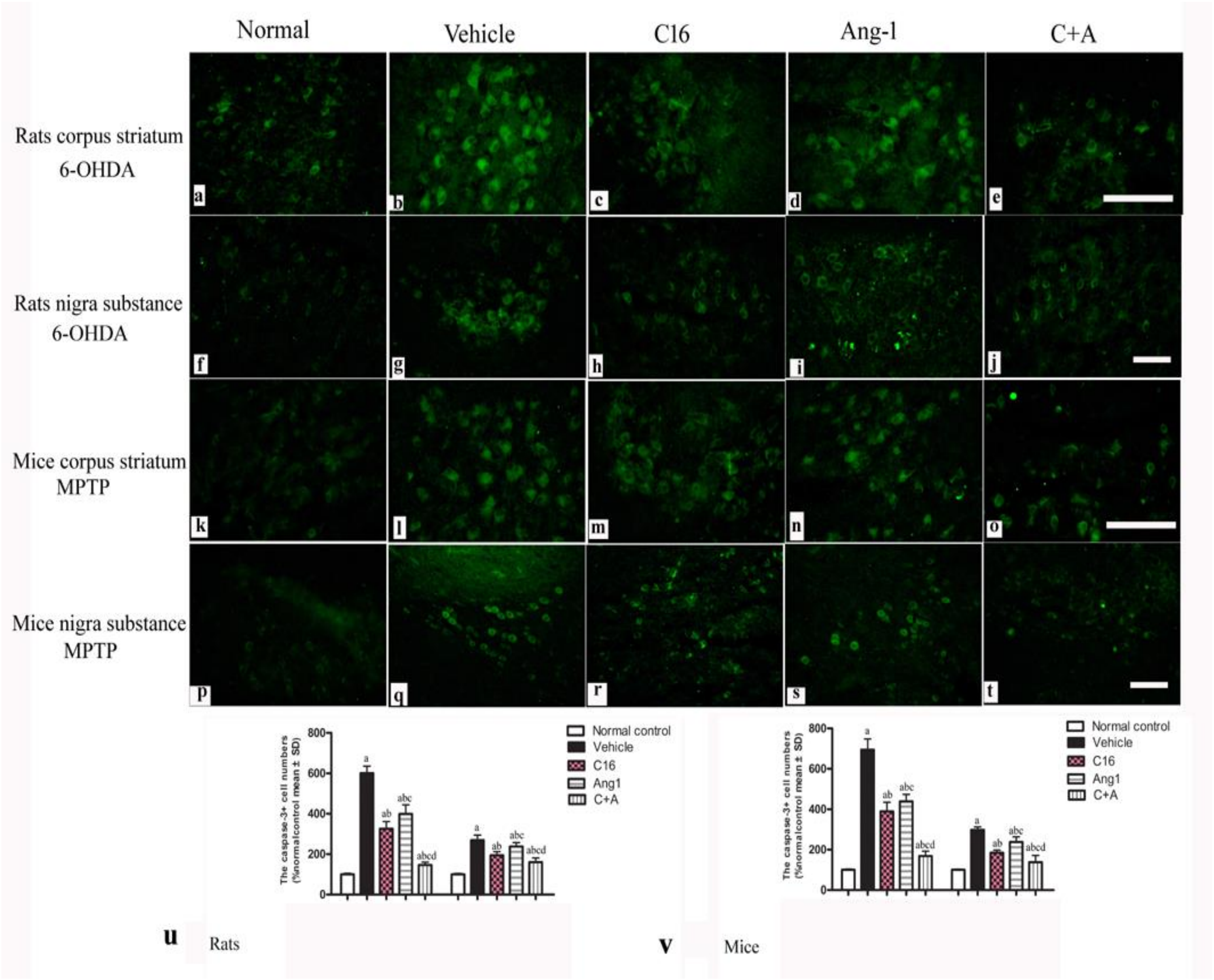

Supplementary Figure 6

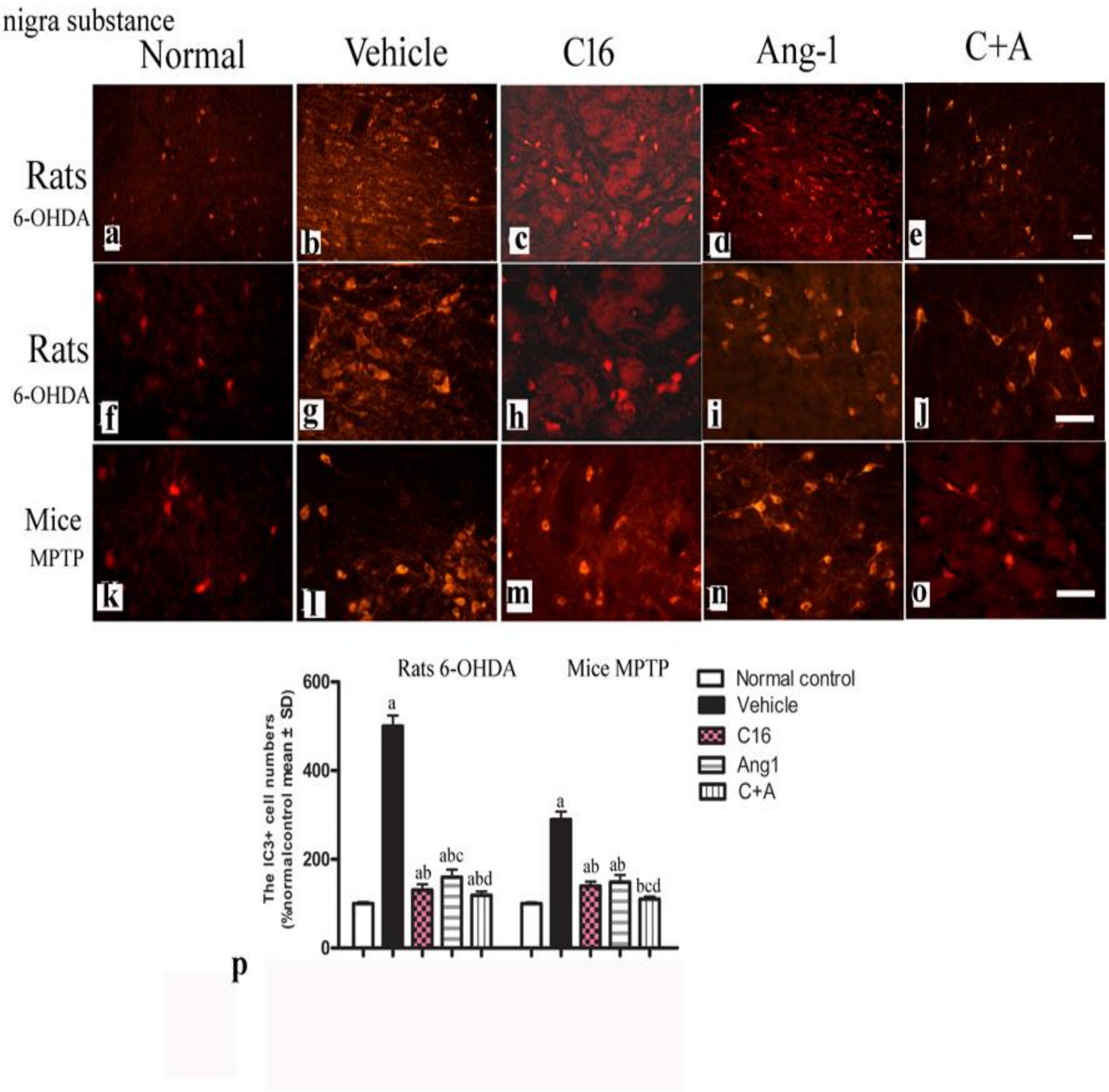

Supplementary Figure 7

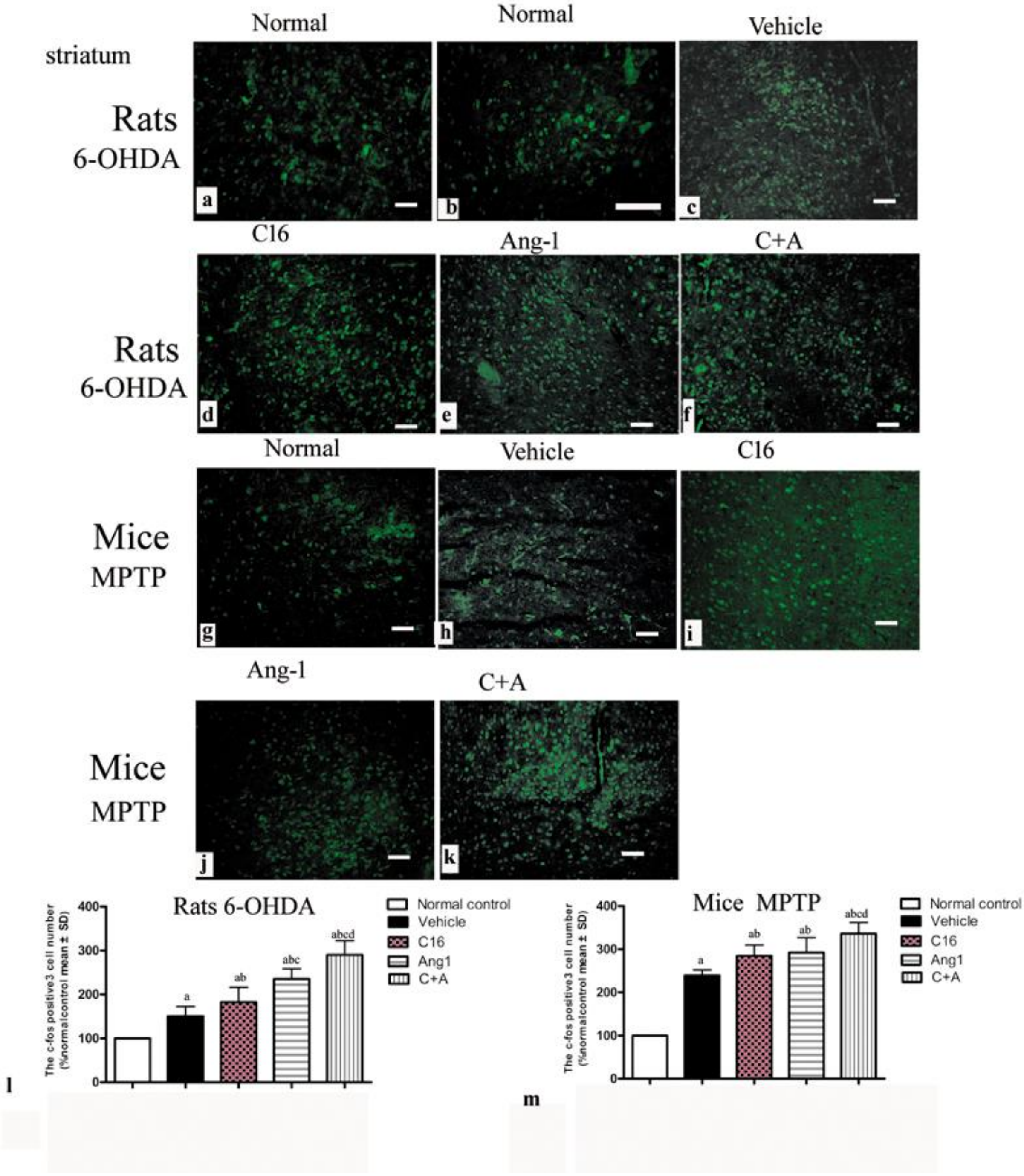

Supplementary Figure 8

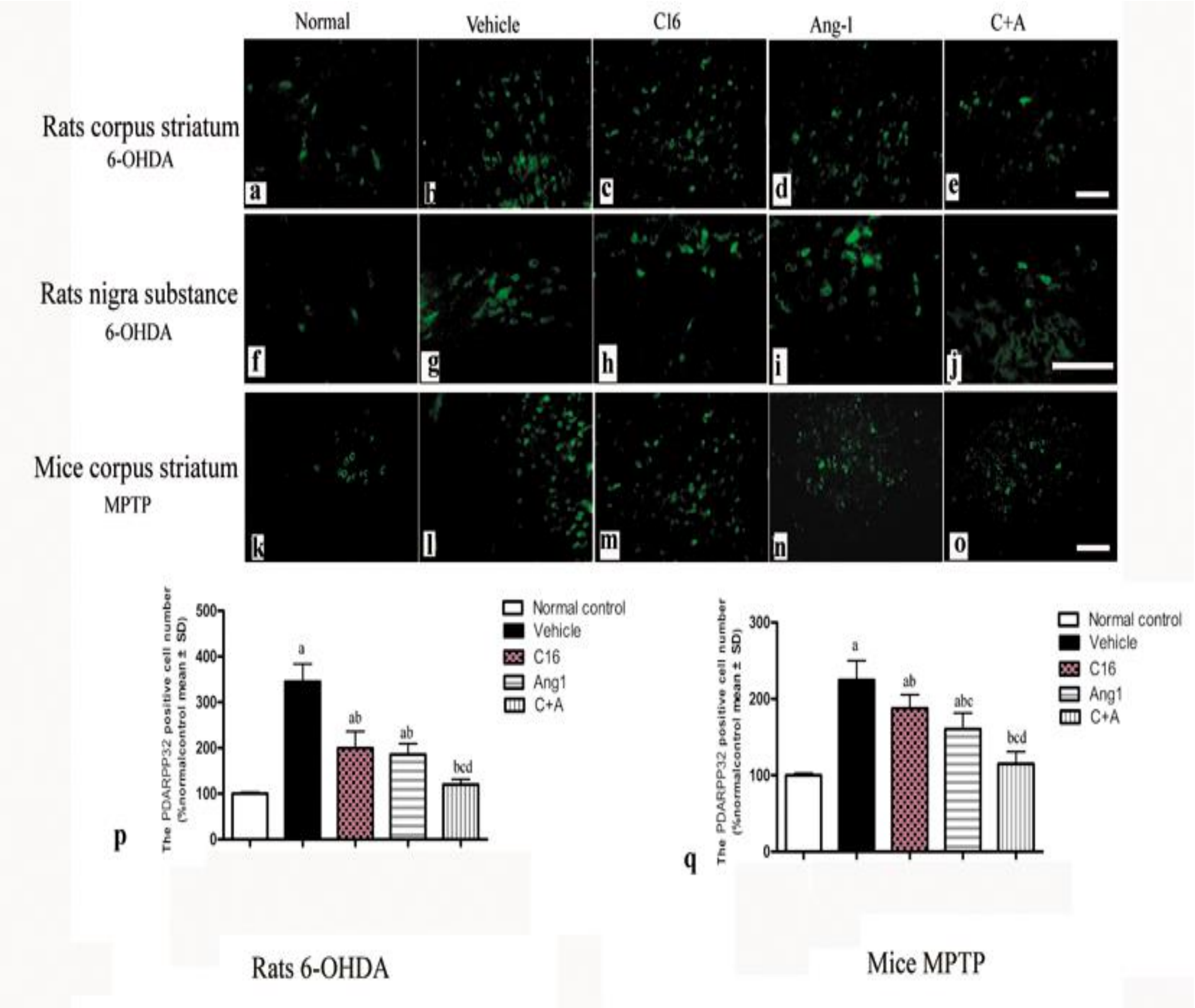

Supplementary Figure 9

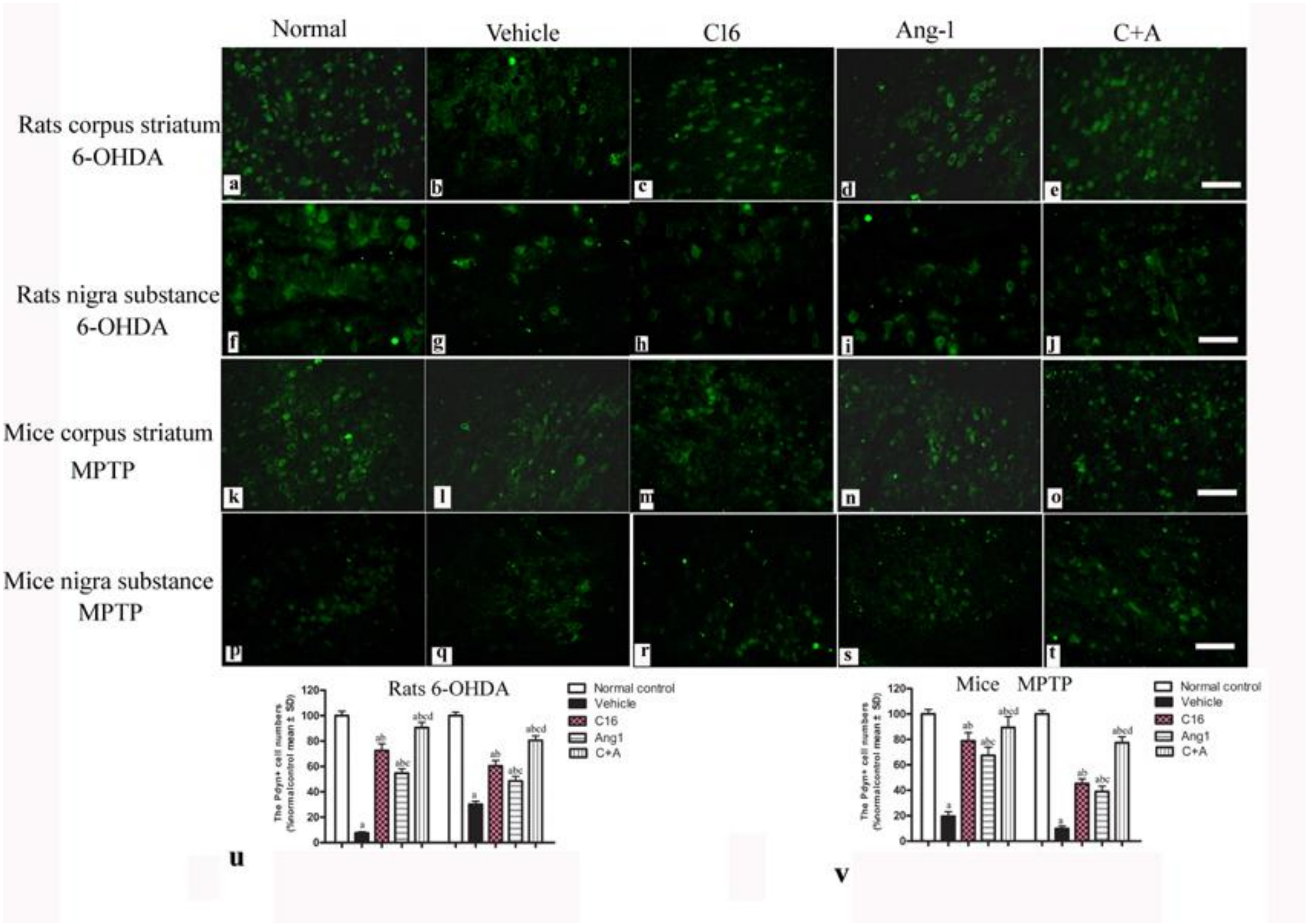

Supplementary Figure 10

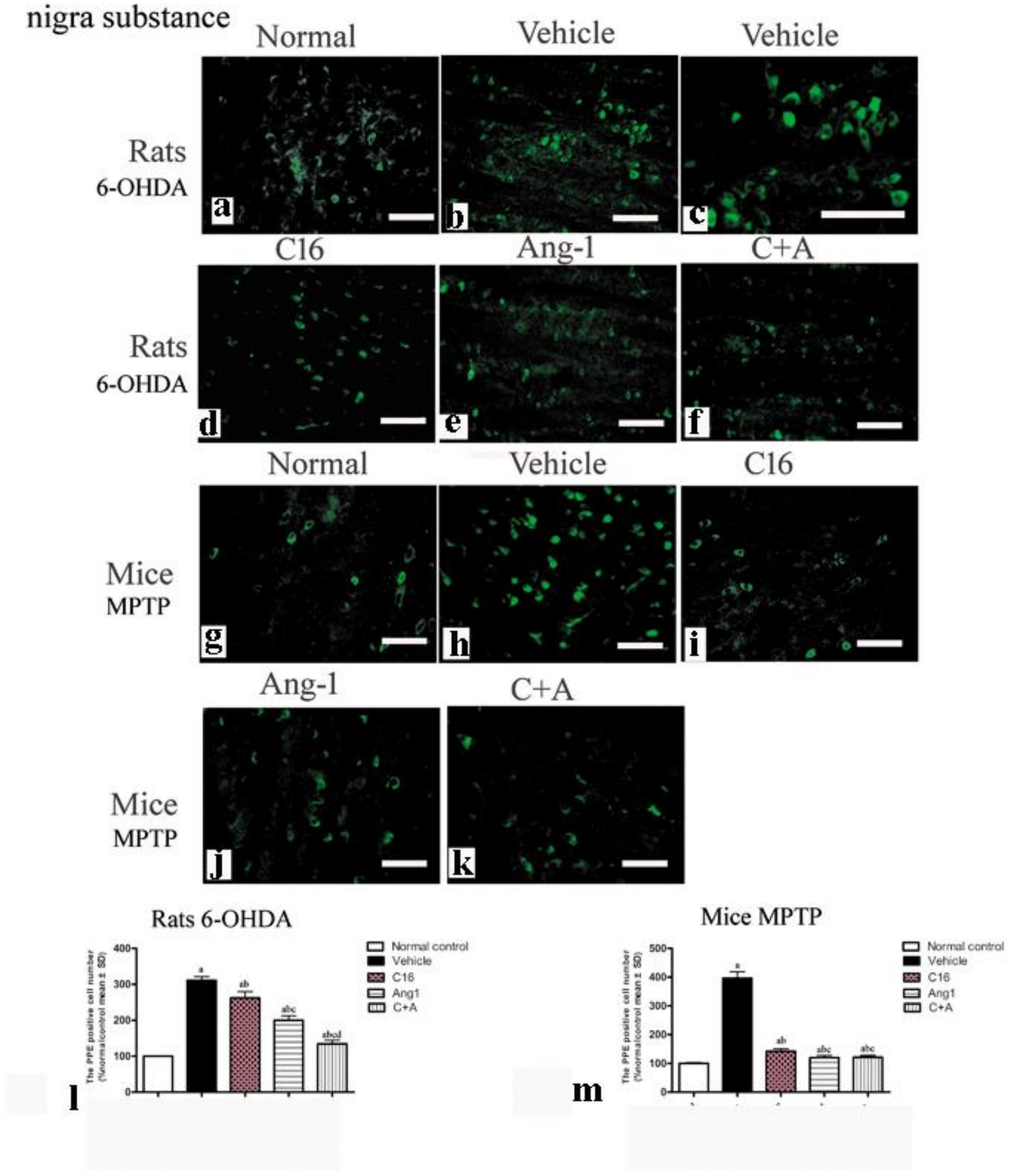

Supplementary Figure 11

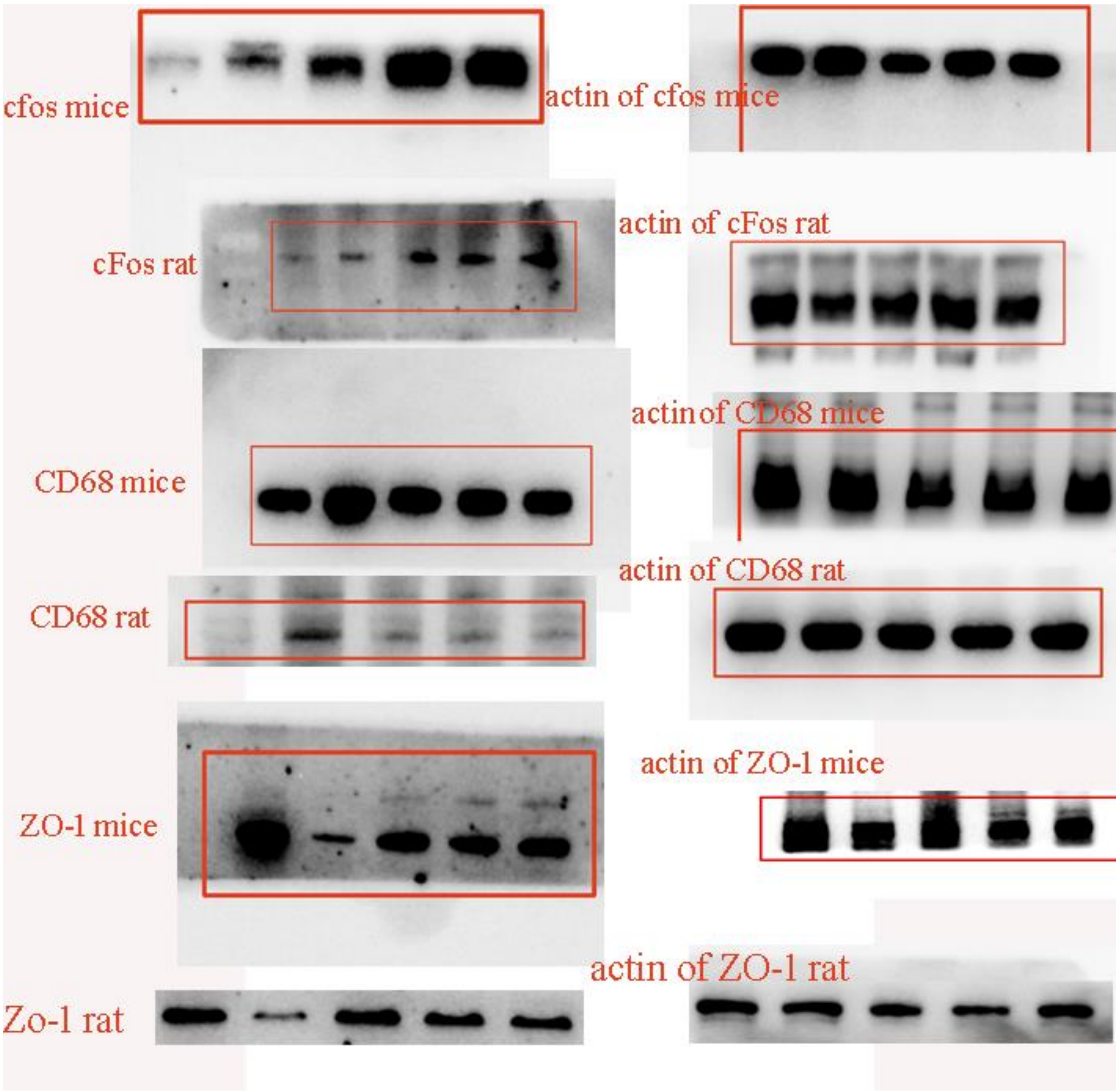

Supplementary Figure 12

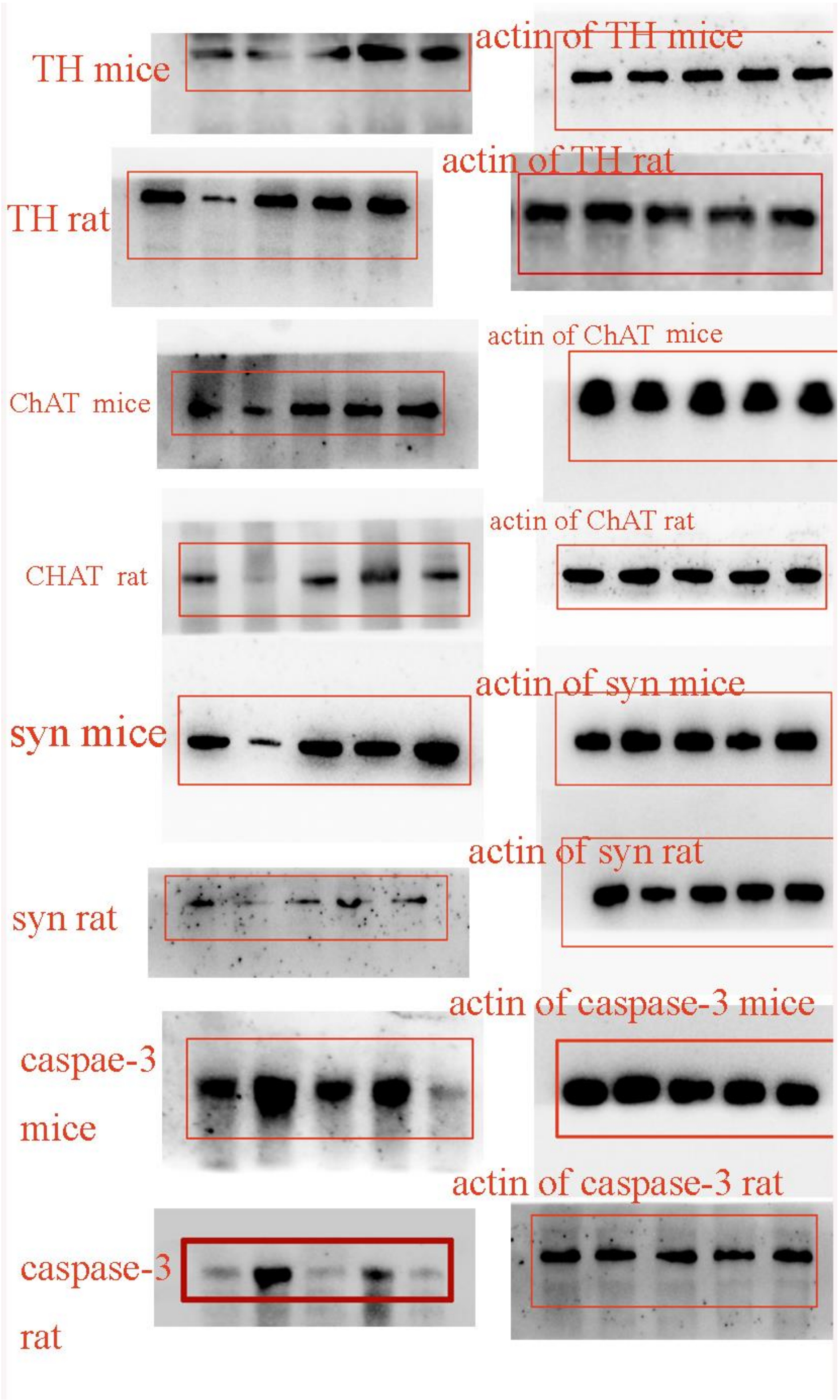

Supplementary Figure 13

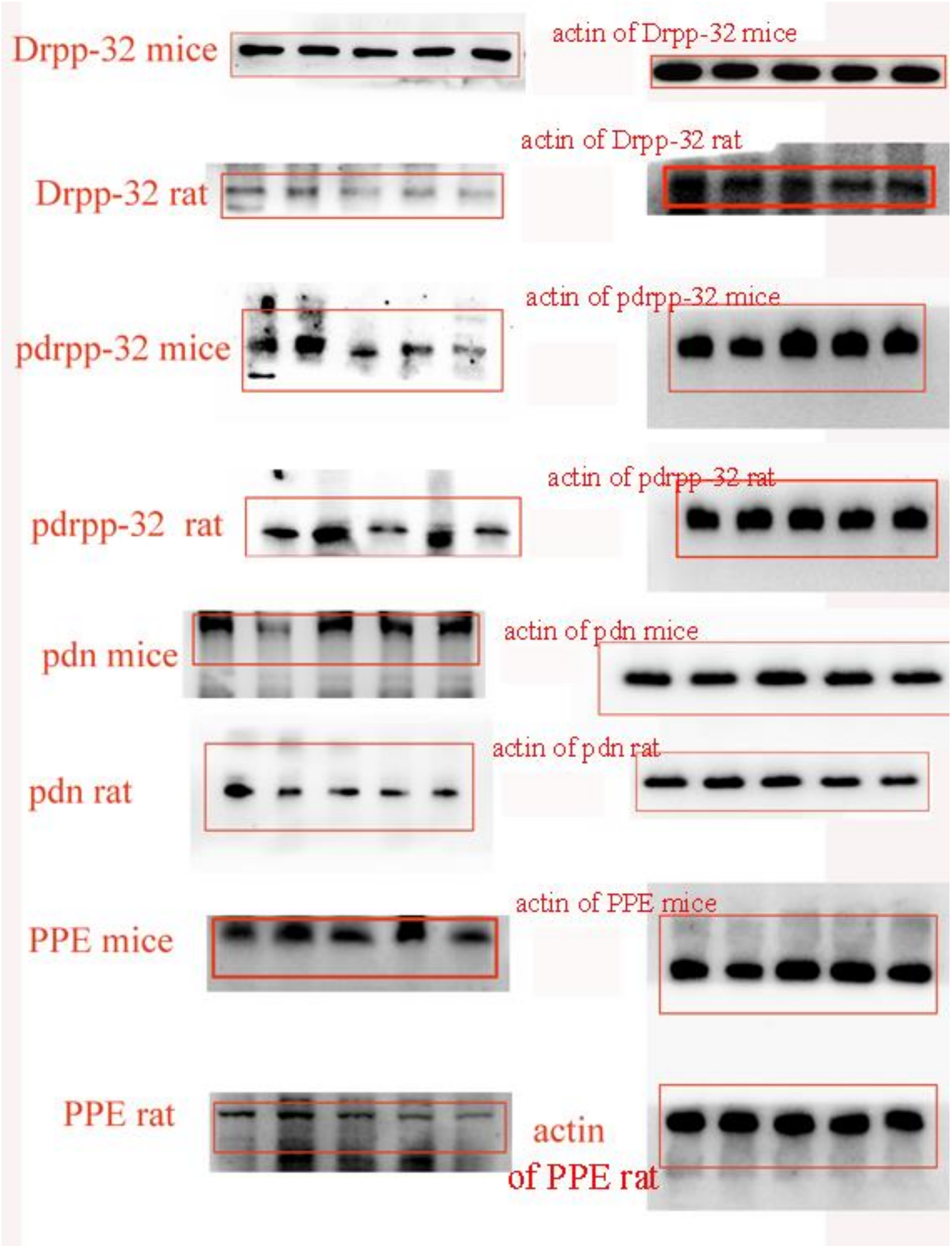

Supplementary Figure 14

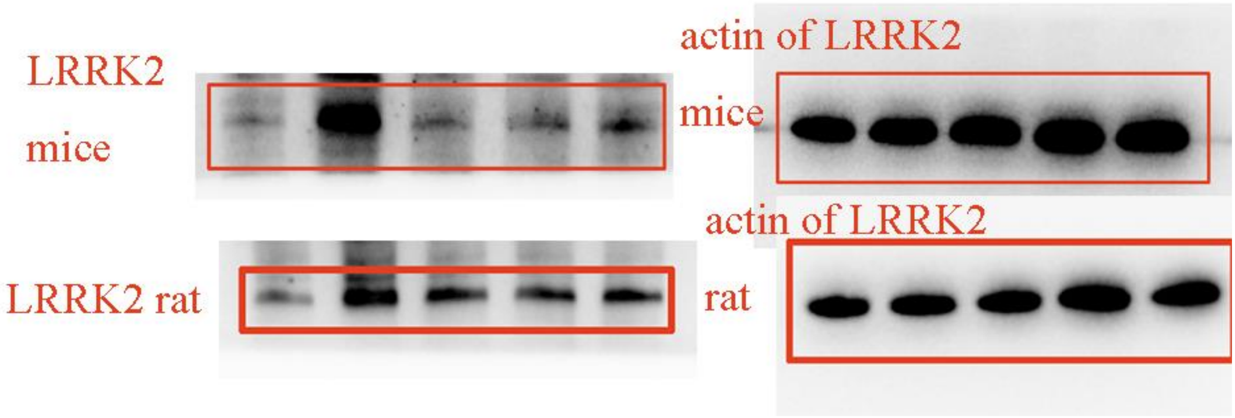

Supplementary Figure 15

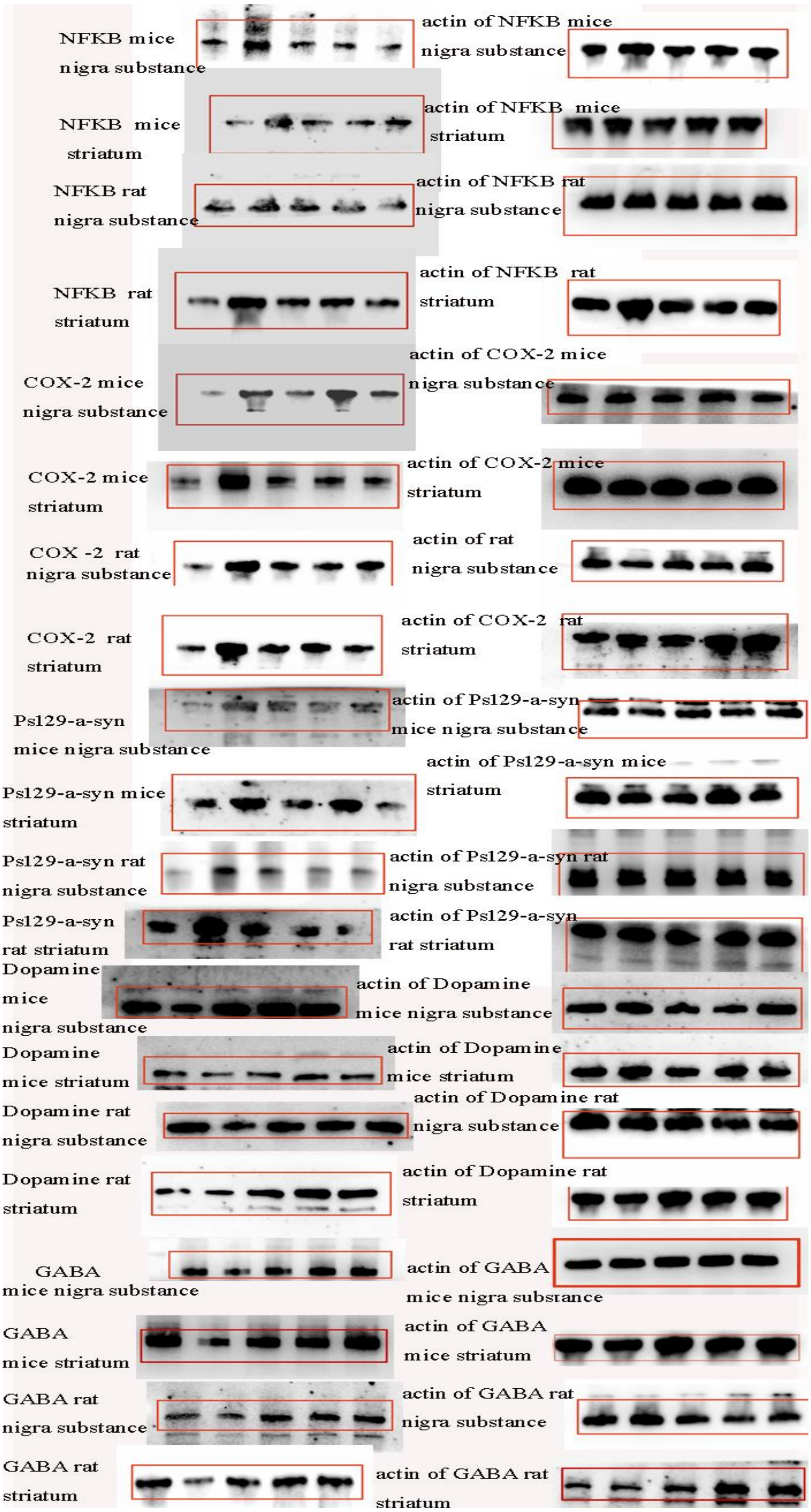

Supplementary Figure 16
